# Supplementary material for: Exploring the genomic resources of seven domestic Bactrian camel populations in China through restriction site-associated DNA sequencing
Source: PLoS One. 2021 Apr 29;16(4):e0250168. doi: 10.1371/journal.pone.0250168 (PMC8084232; doi:10.1371/journal.pone.0250168)
Supplement: S1 Table — (DOCX) [file pone.0250168.s001.docx]

**S1 Table. Sequencing results and quality filtering of reads.**

| **Sample ID** | **Raw data (bp)** | **Clean data (bp)** | **Q20 (%)** | **Q30 (%)** | **GC (%)** | **Number of clean reads** |
| --- | --- | --- | --- | --- | --- | --- |
| **NJ-1** | 1,923,714,618 | 1,888,444,546 | 94.54 | 87.72 | 41.86 | 12,849,068 |
| **NJ-2** | 2,256,475,284 | 2,217,140,920 | 94.66 | 87.97 | 41.86 | 15,085,772 |
| **NJ-3** | 1,669,436,744 | 1,638,753,132 | 94.54 | 87.77 | 41.79 | 11,075,836 |
| **NJ-4** | 2,338,084,760 | 2,296,297,744 | 94.67 | 88.00 | 41.56 | 15,519,716 |
| **NJ-5** | 2,783,092,640 | 2,732,847,981 | 94.65 | 87.98 | 41.45 | 18,469,790 |
| **NJ-6** | 3,388,941,710 | 3,330,190,644 | 94.76 | 88.19 | 40.96 | 22,582,460 |
| **NJ-7** | 1,853,499,541 | 1,818,839,943 | 94.49 | 87.65 | 41.96 | 12,418,044 |
| **BJ-1** | 2,527,717,739 | 2,475,830,840 | 94.36 | 87.45 | 40.79 | 16,903,804 |
| **BJ-2** | 2,436,141,468 | 2,393,113,567 | 94.63 | 87.91 | 41.47 | 16,339,060 |
| **BJ-3** | 2,279,005,980 | 2,235,896,582 | 94.49 | 87.65 | 41.65 | 15,318,494 |
| **BJ-4** | 2,442,658,840 | 2,397,555,661 | 94.61 | 87.94 | 40.77 | 16,425,076 |
| **BJ-5** | 2,034,006,592 | 1,997,661,570 | 94.62 | 87.92 | 41.34 | 13,685,584 |
| **BJ-6** | 1,921,363,504 | 1,887,000,434 | 94.48 | 87.62 | 42.05 | 12,927,946 |
| **BJ-7** | 1,361,748,132 | 1,337,196,198 | 94.52 | 87.69 | 41.76 | 9,161,260 |
| **DJ-1** | 1,904,026,880 | 1,871,441,712 | 94.68 | 88.03 | 41.35 | 12,822,720 |
| **DJ-2** | 3,121,809,012 | 3,089,768,436 | 97.14 | 93.23 | 41.58 | 21,023,556 |
| **DJ-3** | 1,818,269,754 | 1,800,523,106 | 97.17 | 93.28 | 41.54 | 12,251,464 |
| **DJ-4** | 2,869,579,944 | 2,842,384,026 | 97.17 | 93.29 | 42.68 | 19,341,436 |
| **DJ-5** | 4,567,389,695 | 4,304,910,992 | 95.98 | 89.53 | 40.71 | 30,513,168 |
| **DJ-6** | 3,507,693,832 | 3,473,079,253 | 97.15 | 93.22 | 42.85 | 23,474,096 |
| **DJ-7** | 3,261,057,752 | 3,228,334,579 | 97.12 | 93.17 | 42.83 | 21,820,692 |
| **HX-1** | 3,813,080,910 | 3,777,221,567 | 97.25 | 93.47 | 41.31 | 25,615,040 |
| **HX-2** | 3,520,284,832 | 3,485,153,272 | 97.15 | 93.25 | 41.83 | 23,795,788 |
| **HX-3** | 3,077,491,219 | 3,045,289,598 | 97.08 | 93.09 | 41.40 | 20,792,896 |
| **HX-4** | 3,454,365,985 | 3,421,343,897 | 97.20 | 93.35 | 41.68 | 23,360,230 |
| **HX-5** | 3,185,593,564 | 3,154,279,929 | 97.12 | 93.07 | 45.13 | 21,609,186 |
| **QH-1** | 3,566,197,068 | 3,523,728,005 | 96.83 | 92.58 | 41.83 | 23,977,968 |
| **QH-2** | 2,964,245,232 | 2,926,920,051 | 96.80 | 92.55 | 41.67 | 19,784,372 |
| **QH-3** | 3,921,322,752 | 3,872,581,201 | 96.82 | 92.55 | 41.72 | 26,174,636 |
| **QH-4** | 2,849,560,920 | 2,813,725,542 | 96.79 | 92.48 | 41.83 | 19,017,814 |
| **QH-5** | 3,041,945,600 | 3,005,488,473 | 96.82 | 92.52 | 42.71 | 20,384,078 |
| **QH-6** | 2,711,652,884 | 2,677,753,927 | 96.78 | 92.48 | 41.43 | 18,284,078 |
| **QH-7** | 3,031,866,138 | 2,992,531,538 | 96.70 | 92.30 | 41.49 | 20,432,936 |
| **ALS-1** | 3,073,842,536 | 3,043,333,590 | 97.16 | 93.27 | 42.20 | 20,850,150 |
| **ALS-2** | 3,511,102,900 | 3,477,671,146 | 97.22 | 93.40 | 41.37 | 23,825,024 |
| **ALS-3** | 3,323,513,632 | 3,292,014,703 | 97.18 | 93.31 | 41.68 | 22,553,956 |
| **ALS-4** | 3,178,993,196 | 3,149,054,922 | 97.23 | 93.35 | 42.77 | 21,573,390 |
| **ALS-5** | 3,017,539,972 | 2,989,868,222 | 97.26 | 93.46 | 41.81 | 20,483,570 |
| **ALS-6** | 2,888,820,480 | 2,851,489,760 | 96.76 | 92.43 | 41.52 | 19,402,950 |
| **ALS-7** | 1,544,276,748 | 1,525,212,252 | 96.76 | 92.39 | 42.43 | 10,378,418 |
| **SNT-1** | 3,328,148,617 | 3,288,134,940 | 96.83 | 92.56 | 41.52 | 22,451,738 |
| **SNT-2** | 2,598,194,976 | 2,564,381,067 | 96.76 | 92.43 | 41.34 | 17,568,912 |
| **SNT-3** | 2,409,331,420 | 2,379,650,806 | 96.82 | 92.56 | 41.48 | 16,303,460 |
| **SNT-4** | 2,140,263,056 | 2,114,152,181 | 96.73 | 92.39 | 42.08 | 14,486,496 |
| **SNT-5** | 3,498,269,500 | 3,457,098,448 | 96.83 | 92.55 | 41.44 | 23,684,982 |
| **SNT-6** | 2,988,436,624 | 2,952,583,045 | 96.84 | 92.58 | 41.40 | 20,228,716 |
| **SNT-7** | 2,224,795,596 | 2,199,168,964 | 96.88 | 92.63 | 42.31 | 15,067,196 |
