# Supplementary material for: Exploring the genomic resources of seven domestic Bactrian camel populations in China through restriction site-associated DNA sequencing
Source: PLoS One. 2021 Apr 29;16(4):e0250168. doi: 10.1371/journal.pone.0250168 (PMC8084232; doi:10.1371/journal.pone.0250168)
Supplement: Attachment — Submitted filename: PONE-D-20-20388_reviewer-jlh.pdf [file pone.0250168.s024.pdf]

PLOS ONE

Exploring the genom ic resources of seven domestic Bactrian camel populations in China by RAD-seq

--Manuscript Draft--

**Manuscript Number:**	PONE-D-20-20388
**Article Type:**	Research Article
**Full Title:**	Exploring the genom ic resources of seven domestic Bactrian camel populations in China by RAD-seq
**Short Title:**	Exploring the genomic resources of domestic Bactrian camels in China
**Corresponding Author:**	Zhanjun Ren<br>Northwest Agriculture and Forestry University<br>Yangling, CHINA
**Keywords:**	RAD sequencing, Bactrian camel, Single nucleotide polymorphisms, genomic resources
**Abstract:**	The domestic Bactrian camel is a precious livestock resource in arid desert area. It is a kind of livestock with many economic characters, such as producing camel hair, meat, milk, skin, and transport. It has high economic value. Therefore, it is of great significance to explore the genes related to characters of the domestic Bactrian camel in China at the molecular level. In this study, restriction site associated DNA sequencing (RAD-seq) was used to detect SNP markers of the seven domestic Bactrian camel populations. The filtered SNPs were used for Fixation index (F_{ST}) analysis and principal component analysis (PCA) to find out the population which is phylogenetically distant from domestic Bactrian camels in other populations as the control population. A total of 1,568,087 SNPs were genotyped. F_{ST} and PCA showed that Hexi camels were phylogenetically distant from domestic Bactrian camels in other populations. With Hexi camels as the control population and Nanjiang camels, Sunite camels, Alashan camels, Dongjiang camels, Beijiang camels, Qinghai camels as the selection population, 54, 29, 42, 34, 32, 21 selected genes were obtained, respectively. Gene Ontology (GO) classification and Kyoto Encyclopedia of Genes and Genomes (KEGG) enrichment analysis were performed on the selected genes. The function of the selected genes was further studied by Genecards to find out the genes that may be related to the special characters such as heat resistance and stress resistance of the domestic Bactrian camel. Across all populations, genes involved in the cellular process were the most abundant subcategory under biological process. Cell, cell part, and organelle represented the main proportion of genes under cellular component. Binding and catalytic activity represented the main molecular function. Among the Nanjiang camels, Qinghai camels, and Sunite camels, the selected genes were significantly enriched in RNA transport, PI3K-Akt signaling pathway, Mucin type O-glycan biosynthesis and tight junction pathway. The study also found that the Nanjiang camels (HSPA4L, INTU), Sunite camels (SNAI1), Alashan camels (INO80), and Dongjiang camels (UBE2A) may have genes related to environment and characters. The results of gene function analysis are helpful to understand the genes related to the special characters such as heat resistance and stress resistance of the domestic Bactrian camel in China, and provide the basis for the resource development of this species.
**Order of Authors:**	Chenmiao Liu
	Huiling Chen
	Xuejiao Yang
	Chengdong Zhang
	Zhanjun Ren
**Additional Information:**	
**Question**	**Response**
**Financial Disclosure**	This study was supported by the National Natural Science Foundation of China,

Powered by Editorial Manager® and ProduXion Manager® from Aries Systems Corporation

Enter a financial disclosure statement that describes the sources of funding for the work included in this submission. Review the submission guidelines for detailed requirements. View published research articles from *PLOS ONE* for specific examples.<br><br>This statement is required for submission and **will appear in the published article** if the submission is accepted. Please make sure it is accurate.<br><br>**Unfunded studies**<br>Enter: *The author(s) received no specific funding for this work.*<br><br>**Funded studies**<br>Enter a statement with the following details:<br>• Initials of the authors who received each award<br>• Grant numbers awarded to each author<br>• The full name of each funder<br>• URL of each funder website<br>• Did the sponsors or funders play any role in the study design, data collection and analysis, decision to publish, or preparation of the manuscript?<br>• **NO** - Include this sentence at the end of your statement: *The funders had no role in study design, data collection and analysis, decision to publish, or preparation of the manuscript.*<br>• **YES** - Specify the role(s) played.<br><br>* typeset	31172178. The funders had no role in study design, data collection and analysis, decision to publish, or preparation of the manuscript.
**Competing Interests**<br><br>Use the instructions below to enter a competing interest statement for this submission. On behalf of all authors, disclose any competing interests that could be perceived to bias this work—acknowledging all financial support and any other relevant financial or non-financial competing interests.<br><br>This statement **will appear in the published article** if the submission is accepted. Please make sure it is accurate. View published research articles	The authors have declared that no competing interests exist.

Powered by Editorial Manager® and ProduXion Manager® from Aries Systems Corporation

from *PLOS ONE* for specific examples.<br><br>**NO authors have competing interests**<br><br>Enter: *The authors have declared that no competing interests exist.*<br><br>**Authors with competing interests**<br><br>Enter competing interest details beginning with this statement:<br><br>*I have read the journal's policy and the authors of this manuscript have the following competing interests: [insert competing interests here]*<br><br>* typeset	
**Ethics Statement**<br><br>Enter an ethics statement for this submission. This statement is required if the study involved:<br><br>• Human participants<br>• Human specimens or tissue<br>• Vertebrate animals or cephalopods<br>• Vertebrate embryos or tissues<br>• Field research<br><br>Write "N/A" if the submission does not require an ethics statement.<br><br>General guidance is provided below. Consult the submission guidelines for detailed instructions. **Make sure that all information entered here is included in the Methods section of the manuscript.**	All experimental protocols involved in this study were approved by the Institutional Animal Care and Use Committee of the College of Animal Science and Technology, Northwest A & F University, Shaanxi, China (No. DNX20170604).

Powered by Editorial Manager® and ProduXion Manager® from Aries Systems Corporation

**Format for specific study types**<br><br>**Human Subject Research (involving human participants and/or tissue)**<br>• Give the name of the institutional review board or ethics committee that approved the study<br>• Include the approval number and/or a statement indicating approval of this research<br>• Indicate the form of consent obtained (written/oral) or the reason that consent was not obtained (e.g. the data were analyzed anonymously)<br><br>**Animal Research (involving vertebrate animals, embryos or tissues)**<br>• Provide the name of the Institutional Animal Care and Use Committee (IACUC) or other relevant ethics board that reviewed the study protocol, and indicate whether they approved this research or granted a formal waiver of ethical approval<br>• Include an approval number if one was obtained<br>• If the study involved *non-human primates*, add *additional details* about animal welfare and steps taken to ameliorate suffering<br>• If anesthesia, euthanasia, or any kind of animal sacrifice is part of the study, include briefly which substances and/or methods were applied<br><br>**Field Research**<br><br>Include the following details if this study involves the collection of plant, animal, or other materials from a natural setting:<br>• Field permit number<br>• Name of the institution or relevant body that granted permission	
**Data Availability**<br><br>Authors are required to make all data underlying the findings described fully available, without restriction, and from the time of publication. PLOS allows rare exceptions to address legal and ethical concerns. See the PLOS Data Policy and FAQ for detailed information.	Yes - all data are fully available without restriction

A Data Availability Statement describing where the data can be found is required at submission. Your answers to this question constitute the Data Availability Statement and **will be published in the article**, if accepted.<br><br>**Important:** Stating 'data available on request from the author' is not sufficient. If your data are only available upon request, select 'No' for the first question and explain your exceptional situation in the text box.<br><br>Do the authors confirm that all data underlying the findings described in their manuscript are fully available without restriction?	
**Describe where the data may be found in full sentences. If you are copying our sample text, replace any instances of XXX with the appropriate details.**<br><br>• If the data are **held or will be held in a public repository**, include URLs, accession numbers or DOIs. If this information will only be available after acceptance, indicate this by ticking the box below. For example: *All XXX files are available from the XXX database (accession number(s) XXX, XXX.)*.<br>• If the data are all contained **within the manuscript and/or Supporting Information files**, enter the following: *All relevant data are within the manuscript and its Supporting Information files.*<br>• If neither of these applies but you are able to provide **details of access elsewhere**, with or without limitations, please do so. For example:<br><br>*Data cannot be shared publicly because of [XXX]. Data are available from the XXX Institutional Data Access / Ethics Committee (contact via XXX) for researchers who meet the criteria for access to confidential data.*<br><br>*The data underlying the results presented in the study are available from (include the name of the third party*	The datasets generated for this study are available under the NCBI Bioproject PRJNA522647, Biosample SAMN10948548-SAMN10948594.

and contact information or URL).<br>• This text is appropriate if the data are owned by a third party and authors do not have permission to share the data.<br><br>* typeset	
Additional data availability information:	

Powered by Editorial Manager® and ProduXion Manager® from Aries Systems Corporation

Manuscript Click here to access/download;Manuscript;manuscript.doc

Exploring the genomic resources of seven domestic Bactrian camel populations in China by RAD-seq

Chenmiao Liu, Huiling Chen, Xuejiao Yang, Chengdong Zhang, Zhanjun Ren*

College of Animal Science and Technology, Northwest A&F University, Yangling, shaanxi, China

***Corresponding author**

E-mail: Renzhanjun@nwafu.edu.cn (ZJ R)

Abstract

The domestic Bactrian camel is a precious livestock resource in arid desert area~~. It is a kind of livestock with many economic characters, such as~~ producing camel hair, meat, milk, skin, and transport. ~~It has high economic value.~~ Therefore, it is of great significance to explore the genes related to characters of the domestic Bactrian camel~~ in China at the molecular level~~. In this study, restriction site associated DNA sequencing (RAD-seq) was used to detect SNP markers of the seven domestic Bactrian camel populations. The filtered SNPs were used for ~~F~~ixation index (F_{ST}) ~~analysis~~ and principal component analysis (PCA) to find out the population which is phylogenetically distant from domestic Bactrian camels in other populations as the control population. A total of 1,568,087 SNPs were genotyped. F_{ST} and PCA showed that Hexi camels were phylogenetically distant from domestic Bactrian camels in other

populations. With Hexi camels as the control population ~~and~~ Nanjiang camels, Sunite camels, Alashan camels, Dongjiang camels, Beijiang camels, Qinghai camels as the selection population, 54, 29, 42, 34, 32, 21 selected genes were obtained, respectively. Gene Ontology (GO) classification and Kyoto Encyclopedia of Genes and Genomes (KEGG) enrichment analysis were performed on the selected genes. The function of the selected genes was further studied by Genecards to find out the genes that may be related to the special characters such as heat resistance and stress resistance of the domestic Bactrian camel. Across all populations, genes involved in the cellular process were the most abundant subcategory under biological process. Cell, cell part, and organelle represented the main proportion of genes under cellular component. Binding and catalytic activity represented the main molecular function. Among the Nanjiang camels, Qinghai camels, and Sunite camels, the selected genes were significantly enriched in RNA transport, PI3K-Akt signaling pathway, Mucin type O-glycan biosynthesis and tight junction pathway. The study also found that the Nanjiang camels (*HSPA4L*, *INTU*), Sunite camels (*SNAI1*), Alashan camels (*INO80*), and Dongjiang camels (*UBE2A*) may have genes related to environment and characters. The results of gene function analysis are helpful to understand the genes related to the special characters such as heat resistance and

stress resistance of the domestic Bactrian camel in China, and provide the basis for the resource development of this species.

Introduction

The domestic Bactrian camel is not only an important livestock resource in China, an important means of transportation for cultural exchange between the East and the West, but also a kind of livestock with various economic and production characters [1]. Camel milk is rich in nutrition, with hypoglycemic and anticancer activities [2, 3]. Camel hair is the best textile material and is called "natural protein fiber" by fiber experts [4]. Camel meat not only has the characteristics of high moisture, high protein, low fat and low cholesterol, but also has medicinal and health care value, and can be used to treat related diseases [5, 6, 7]. In addition, the domestic Bactrian camel lives in hot and arid desert environment, which has potential ecological adaptation value. Therefore, it is necessary to excavate the genomic resources. Felkel et al. [8] (2019) generated a 3.8 Mbp sized assembly of the MSY of ~~Camelus bactrianus~~ based on short read next generation sequencing data. Based on 596 single nucleotide variants, the Y phylogenetic network with seven haplotypes has been revealed, and the divergence time of wild and domestic Bactrian camels was estimated. In the previous study, we used SNP to study the genetic diversity and genetic structure of seven domestic Bactrian camel

populations, clarified their phylogenetic relationship, and laid the foundation for protecting their biodiversity [9].

RAD-seq technology is a simplified genome sequencing technology based on the whole genome restriction sites developed on the basis of the second generation sequencing. With the development of high-throughput sequencing technology and bioinformatics technology, RAD-seq analysis is becoming more and more perfect, and has been applied to many organisms [10]. Barchi et al. [11] developed more than 1,000 SNP markers of eggplant (*Solanum melongena*) using RAD-seq technology for marker-assisted selection and comparative genomics analysis. Using RAD-seq, Li et al. [12] analyzed the genetic diversity and genetic structure of the Bian chicken conservation population (*Gallus gallus*), provided the basis for formulating scientific conservation measures and diversified conservation methods.

In the study, RAD-seq was used to detect SNP markers of seven domestic Bactrian camel populations. F_{ST} and PCA were used to find out the population, which is phylogenetically distant from domestic Bactrian camels in other populations as the control population. The selected genes of all populations were identified by analyzing selection signals. GO and KEGG analysis were performed on the identified selected genes, which was further studied by Genecards to find out the genes that may be related to the special characters such as heat resistance and stress

resistance of the domestic Bactrian camel. The study explored the genomic resources of the domestic Bactrian camel in China, and provided a reference for the protection and utilization of this species.

Materials and methods

Animals and sampling sites

The domestic Bactrian camels in China mainly inhabit the arid desert and semi-desert areas in Northwest China. The domestic Bactrian agricultural camels were sampled at seven sites in Inner Mongolia, Gansu, Qinghai and Xinjiang. A total of forty-seven venous blood samples (Table 1) were collected to ensure that each sample was derived from a different family, and there was no kinship between individuals. All experimental protocols involved in this study were approved by the Institutional Animal Care and Use Committee of the College of Animal Science and Technology, Northwest A & F University, Shaanxi, China (No. DNX20170604).

Table 1. Information for domestic Bactrian camels in the seven sampled locations of China.

**Code**	**Population**	**Location**	**Latitude**	**Longitude**	**Number**
**NJ**	Nanjiang camel	Wensu county, Xinjiang	41°29′	80°24′	7
**BJ**	Beijiang camel	Qinghe county, Xinjiang	46°71′	90°37′	7
**DJ**	Dongjiang camel	Mulei county, Xinjiang	43°80′	90°34′	7
**HX**	Hexi camel	Yongchang county, Gansu	38°25′	101°97′	5
**QH**	Qinghai camel	Mohe, Qinghai	36°77′	99°05′	7
**ALS**	Alashan camel	Alashan Left Banner, Inner Mongolia	38°85′	105°68′	7
**SNT**	Sunite camel	Sunite Right Banner, Inner Mongolia	42°47′	112°95′	7
**Total**					47

DNA extraction, library construction, and Illumina sequencing

DNA samples were extracted following a standard phenol-chloroform extraction procedure (Sambrook and Russell, 2002) and were diluted to 20 ng/μL [13]. Then, the quality and quantity of DNA were evaluated by Epoch spectrophotometer (BioTek, Winooski, USA), agarose gel electrophoresis, and Qubit 3.0 fluorometer (Thermo Fisher Scientific, Waltham, USA). The DNA samples were further processed [14].

RAD-seq libraries were constructed in accordance with the modified protocol [15]. First, EcoRI (New England Biolabs) was used to digest genomic DNA (0.1 – 1 μg; from a single sample or pooled samples), and P1 adaptors were connected at the cutting site. Then, the samples were pooled, randomly sheared, and size-selected in sequential steps. After the P2 adaptors were added, the sequencing libraries were constructed using DNA fragments of 300-700 bp in length. Finally, on the Illumina HiSeq3000 platform, the samples were sequenced using 100 bp paired -end reads.

Quality control, read mapping, and SNP calling

This filtering step of reads was performed using the fastp tool (v0.19.5) [16]. Applying three strict filtering criteria, raw reads were processed to obtain high-quality clean reads: (i) removing readings with ≥ 10% unidentified nucleotides (N); (ii) removing readings with > 50% bases

having phred quality scores of ≤20; and (iii) removing readings aligned to the barcode adapter.

The Burrows-Wheeler Aligner (BWA) was used to align the clean readings of each sample with the reference genome (https://www.ncbi.nlm.nih.gov/genome/10741) with the settings 'mem 4 -k 32 -M', -k is the minimum seed length, and -M is an option for marking shorter split alignment hits as secondary alignments [17]. GATK's Unified Genotyper was used to conduct variant calling on all samples [18]. SNPs were filtered using the GATK's Variant Filtration with proper standards (-Window 4, -filter "QD < 2.0 || FS > 60.0 || MQ <40.0", -G_filter "GQ < 20").

F_{ST} analysis and Principal component analysis

F_{ST} was calculated by the F_{ST} statistics function in the PopGenome package to study the genetic diversity between different populations [19]. F_{ST} was also used to study the genetic distance between different populations and to express differences of population genetic structure. [20]. The larger the Fst value is, the farther the genetic distance between the two populations is.

PCA (Principal component analysis) converts multiple differences into a few independent factors and analyzes the differences between different populations based on the degree of difference between these factors to further classify different populations [21]. PCA can also be used to study

the genetic distance between populations [22]. In this study, we used the GCTA software in R for PCA to further study the genetic structure between populations [23].

Analyses of selection signals

Selection signals detection used 50 kb as the window and 25 kb as the step for sliding π value and F_{ST} distribution calculation, the -log10 transform of Nei's π was used to select the lower end of diversity windows, and these parameters were quantified by internal PERL Scripts. F_{ST} and π ~~of top 1%~~ were selected as candidate regions [24, 25]. All related graphs were drawn using R scripts [26].

Sequence annotation and enrichment analyses

In order to further systematically elucidate the complex biological functions of the genes, the selected genes of Nanjiang camels, Sunite camels, Alashan camels, Dongjiang camels, Beijiang camels and Qinghai camels were mapped against both GO and KEGG databases. GO enrichment analysis was performed with WEGO software, and gene numbers were calculated for every term [27]. KOBAS software and KEGG database (http://www.genome.jp/kegg/) were used to test the statistical enrichment of selected genes [28]. The calculated p-value was subject to false discovery rate (FDR) correction, applying FDR⩽0.05 as the threshold. Pathways meeting this condition were defined as significantly enriched pathways.

Results

RAD-tag sequencing and data filtering

The raw data of 131G and 893,672,274 reads from the seven populations were obtained by Illumina sequencing. After quality filtering ~~of the sequence data, a total of~~ 129G of clean data and 882,097,022 clean reads were retained, with an average of 2.75G of clean data per sample, and an average of 19.01 million readings per sample, with an average effective rate of 98.57%. Q20 was higher than 94%, Q30 was higher than 87%, and the GC content was stable, at between 40.71% and 45.13% (S1 Table). In short, the sequencing data showed a high phred quality.

F_{ST} analysis and Principal component analysis

The Fst values between different populations were calculated to study the genetic distance between different populations. As shown in Table 2, it can be concluded that the average Fst between the Hexi camels and the domestic Bactrian camels in other populations was 0.2068, which was the highest among all the average values, indicating a large genetic distance between Hexi camels and other domestic Bactrian camels.

Table 2. The F_{ST} values between the populations of domestic Bactrian camels in China.

	BJ	DJ	HX	NJ	QH	SNT	Mean
**ALS**	0.1951	0.1478	0.2023	0.1914	0.1987	0.1651	0.1834
**BJ**		0.1632	0.2258	0.2140	0.2142	0.1826	0.1991
**DJ**			0.1759	0.1622	0.1710	0.1362	0.1594
**HX**				0.2208	0.2246	0.1916	0.2068
**NJ**					0.2141	0.1801	0.1971
**QH**						0.1893	0.2020
**SNT**							0.1742

Based on the first two PCs (PC1=4.13%, PC2=3.97%), the Qinghai camels clustered together, the Hexi camels clustered together, and they were phylogenetically distant from the domestic Bactrian camels in other populations. Based on the two PCs (PC1=4.13%, PC3=3.71%), the Qinghai camels, Hexi camels, Nanjiang camels and Alashan camels clustered together separately, and they were phylogenetically distant from the domestic Bactrian camels in other populations. Based on the last two PCs (PC2=3.97%, PC3=3.71%), the Hexi camels, Nanjiang camels and Alashan camels clustered together separately, and they were phylogenetically distant from the domestic Bactrian camels in other populations. The three PCA figures showed that the Hexi camels clustered together separately, and they were phylogenetically distant from the domestic Bactrian camels in other populations (Fig 1). These findings were consistent with that of Fst analysis. Therefore, we regarded the Hexi camels as a control population for analysis of selection signals.

Fig 1. Principal component analysis of seven domestic Bactrian camel populations in China. (a) Principal components 1 and 2 from principal component analysis (PCA). (b) Principal components 1 and 3 from

principal component analysis (PCA). (c) Principal components 2 and 3 from principal component analysis (PCA). ALS, Alashan camels; BJ, Beijiang camels; DJ, Dongjiang camels; HX, Hexi camels; NJ, Nanjiang camels; QH, Qinghai camels; SNT, Sunite camels

Analyses of selection signals

F_{ST} and π were used to select the top 1% regions. With Hexi camels as the control population and Nanjiang camels, Sunite camels, Alashan camels, Dongjiang camels, Beijiang camels and Qinghai camels as the selection population, 54, 29, 42, 34, 32, 21 selected genes were obtained (Fig 2-5, Fig S1-S2). The selected genes in the scanning regions were extracted and the function of the selected genes was predicted by Genecards (http://www.genecards.org/). The analysis indicates that the Nanjiang camels (*HSPA4L*, *INTU*), Sunite camels (*SNAI1*), Alashan camels (*INO80*), and Dongjiang camels (*UBE2A*) may have genes related to environment and characters.

Fig 2. GO classification and KEGG enrichment of the selected genes of Nanjiang camels. (a) With Hexi camels as the control population and Nanjiang camels as the selection population, 54 selected genes were obtained. (b) KEGG enrichment of 54 selected genes of Nanjiang camels. (c) GO classification of 54 selected genes of Nanjiang camels.

Fig 3. GO classification and KEGG enrichment of the selected genes of Sunite camels. (a) With Hexi camels as the control population and

Sunite camels as the selection population, 29 selected genes were obtained. (b) KEGG enrichment of 29 selected genes of Sunite camels. (c) GO classification of 29 selected genes of Sunite camels.

Fig 4. GO classification and KEGG enrichment of the selected genes of Alashan camels. (a) With Hexi camels as the control population and Alashan camels as the selection population, 42 selected genes were obtained. (b) KEGG enrichment of 42 selected genes of Alashan camels. (c) GO classification of 42 selected genes of Alashan camels.

Fig 5. GO classification and KEGG enrichment of the selected genes of Dongjiang camels. (a) With Hexi camels as the control population and Alashan camels as the selection population, 34 selected genes were obtained. (b) KEGG enrichment of 34 selected genes of Dongjiang camels. (c) GO classification of 34 selected genes of Dongjiang camels.

Sequence annotation and enrichment analysis

GO classification was carried out on the selected genes. Across all populations, genes involved in the cellular process (GO:0009987) were the most abundant subcategory under biological process. Cell (GO:0005623), cell part (GO:0044464), and organelle (GO:0043226) represented the main proportion of genes under cellular component. Binding (GO:0005488) and catalytic activity (GO:0003824) represented the main molecular function (Fig 2-5, Fig S1-S2).

KEGG enrichment analysis was carried out on the selected genes of six domestic Bactrian camel populations. In the Alashan camels, the selected genes were mainly enriched in metabolic pathways. In the Dongjiang camels, the selected genes were mainly enriched in endocytosis pathways. In the Nanjiang camels, the selected genes were significantly enriched in RNA transport pathways, mainly in RNA transport pathways and metabolic pathways. In the Qinghai camels, the selected genes were significantly enriched in PI3K-Akt signaling pathways and Mucin type O-glycan biosynthesis pathways, mainly in PI3K-Akt signaling pathways. In the Sunite camels, the selected genes were significantly enriched in tight junction pathways, mainly in tight junction pathways.

Discussion

In the study, a total of 1,568,087 SNP markers in seven domestic Bactrian camel populations were detected by RAD-seq. The filtered SNPs were used for Fst and PCA to find out the population which is phylogenetically distant from the domestic Bactrian camels in other populations as the control population. The results showed that the Hexi camels clustered together separately and were phylogenetically distant from the domestic Bactrian camels in other populations, which was consistent with the existing literature [9]. This may be related to the geographic location of the Hexi camels. Yongchang County is located in the northern foot of the Qilian Mountains, close to the Badain Jaran desert, resulting in less

communication between the Hexi camels and other domestic Bactrian camel populations. With Hexi camels as the control population and Nanjiang camels, Sunite camels, Alashan camels, Dongjiang camels, Beijiang camels, Qinghai camels as the selection population for analysis of selection signals, 54, 29, 42, 34, 32, 21 selected genes were obtained respectively. GO classification and KEGG enrichment analysis were performed on the selected genes. Using Genecards to further study the function of the selected genes, we found that the Nanjiang camels (HSPA4L, INTU), Sunite camels (SNAI1), Alashan camels (INO80), and Dongjiang camels (UBE2A) may have genes related to environment and characters. This finding fills the gap in the genome study of the domestic bactrian camel in China [29, 30, 31].

HSPA4L (Heat Shock Protein Family A (Hsp70) Member 4 Like) is a protein coding gene. Its related pathway is cellular response to heat stress, which is a candidate gene related to heat resistance [29]. Wensu county is close to the Taklimakan Desert. Nanjiang camels have been living in the natural environment with abundant light energy, dry and little rain, strong wind and sand for a long time, and have extremely strong heat resistance [32, 33]. It can be concluded that *HSPA4L* is an interesting target for studying the heat resistance of Nanjiang camels. The Nanjiang camels have 3cm-5cm long eyelashes. Long eyelashes can protect the eyes from strong sunlight and prevent sand and other foreign matters from entering

under the condition of sandstorm. The length of the eyelashes is determined by the duration of its hair follicle growth period [34]. *INTU* (Inturned Planar Cell Polarity Protein) is involved in hair follicle morphogenesis [35]. Therefore, *INTU* is an interesting target for studying the eyelashes length of Nanjiang camels.

Sunite camels have solid bones, strong muscles, thick and dense villi layer and many protective hairs. It is the best breed of the domestic Bactrian camels in China with the largest body shape, villi yield and meat yield [36]. The key to increase the villi yield is to ensure the full development of hair follicle [37]. *SNAI1* (Snail Family Transcriptional Repressor 1) is involved in hair follicle morphogenesis [38]. Therefore, *SNAI1* is an interesting target for studying the villi yield of Sunite camels.

The central production area of Alashan camels is located in the Alashan Plateau in the west of Inner Mongolia. The climate of Alashan Plateau is dry and the ultraviolet ray is strong. Alashan camels have formed unique physiological function and resistance during the long-term evolution process, such as drought resistance and UV resistance [39]. *INO80* (*INO80* Complex ATPase Subunit) is involved in cellular response to UV [40]. *INO80* is therefore an interesting target for future validation.

The central production area of Dongjiang camels is located in Mulei county, which is located in the temperate desert with strong ultraviolet rays and harsh natural ecological conditions [41]. *UBE2A* (Ubiquitin

Conjugating Enzyme E2A) is involved in cellular response to UV [42]. Therefore, *UBE2A* is an interesting target for future study.

In brief, the SNP loci of seven domestic Bactrian camel populations in China were developed at the whole genome level by RAD-seq. The selected gene was identified by analyses of selection signals. The study also found that the Nanjiang camels, Sunite camels, Alashan camels, and Dongjiang camels may have genes related to environment and characters. It has laid a foundation for further excavation of genomic resources of the domestic Bactrian camel in China, which will help us better protect and develop the genetic resources of this species. In the next step of research, we will deeply excavate the genes related to the characters, and explore the available genomic resources of this species.

Supporting information

S1 Table. Sequencing results and quality filtering of reads. (DOCX)

S1 Fig. GO classification and KEGG enrichment of the selected genes of Beijiang camels. (a) With Hexi camels as the control population and Beijiang camels as the selection population, 32 selected genes were obtained. (b) KEGG enrichment of 32 selected genes of Beijiang camels. (c) GO classification of 32 selected genes of Beijiang camels. (TIF)

S2 Fig. GO classification and KEGG enrichment of the selected genes of Qinghai camels. (a) With Hexi camels as the control population and Qinghai camels as the selection population, 21 selected genes were

obtained. (b) KEGG enrichment of 21 selected genes of Qinghai camels. (c) GO classification of 21 selected genes of Qinghai camels. (TIF)

Author Contributions

Conceptualization: Chenmiao Liu, Zhanjun Ren.

Data curation: Xuejiao Yang, Chengdong Zhang.

Resources: Zhanjun Ren, Huiling Chen.

Writing-original draft: Chenmiao Liu.

References

1. Wang Q, Zhang Q, Gan Z, Li H, Yang Y, Zhang Y, et al. Screening for reproductive biomarkers in Bactrian camel via iTRAQ analysis of proteomes. Reproduction in domestic animals. 2019; 1-11. doi: 10.1111/rda.13607.

2. Krishnankutty R, Iskandarani A, Therachiyil L, Uddin S, Azizi F, Kulinski M, et al. Anticancer activity of camel milk via induction of autophagic death in human colorectal and breast cancer cells. Asian pacific journal of cancer prevention. 2018;19(12): 3501-3509. doi: 10.31557/APJCP.2018.19.12.3501.

3. Lu DL, Xu M, Li JF, He XR. Health care function and auxiliary effect of camel milk. Xinjiang animal husbandry. 2019;34(4): 17-23. doi: 10.16795/j.cnki.xjxmy.2019.4.001.

4. Wang HB, Gao YQ, Li WH, Liang LN, Chang YL. Research status of camel production performance and camel hair quality. Contemporary

animal husbandry. 2007;7: 50-51.

5. Yang L, Fu YH, Zhang ZX, Feng XH, Yang J. Current status of nutritional value, meat quality and processing of camel meat. Meat research. 2018;32(6): 55-60.

6. Bekhit AE, Farouk MM, Kadim IT. Nutritive and heath value of camel meat. Wallingford: Center for agriculture and bioscience international. 2013; 205-223. doi: 10.1079/9781780641010.0205.

7. Abdelhadi OMA, Babiker SA, Bauchart D, Listrat A, Rémond D, Hocquette JF, et al. Effect of gender on quality and nutritive value of dromedary camel (Camelus dromedarius) longissimus lumborum muscle. Journal of the Saudi Society of Agricultural Sciences. 2017;16(3): 242-249. doi: 10.1016/j.jssas.2015.08.003.

8. Felkel S, Wallner B, Chuluunbat B, Yadamsuren A, Faye B, Brem G, et al. A first Y-chromosomal haplotype network to investigate male-driven population dynamics in domestic and wild Bactrian camels. Frontiers in genetics. 2019;10: 1664-8021. doi: 10.3389/fgene.2019.00423.

9. Liu CM, Chen HL, Ren ZJ, Zhang CD, Yang XJ. Population genetic analysis of the domestic Bactrian camel in China by RAD-seq. Ecology and evolution. 2019;34(4): 11232-11242. doi: 10.1002/ece3.5624.

10. Basak M, Uzun B, Yol E. Genetic diversity and population structure of the Mediterranean sesame core collection with use of genome-wide SNPs developed by double digest RAD-Seq. PLoS One. 2019;14(10):

e0223757. doi: 10.1371/journal.pone.0223757.

11. Barchi L, Lanteri S, Portis E, Acquadro A, Valè G, Toppino L, et al. Identification of SNP and SSR markers in eggplant using RAD tag sequencing. BMC genomics. 2011;12(1): 304. doi: 10.1186/1471-2164-12-304.

12. Li GH, Wei QY, Zhang HY, Yin JM, Xue Q, Zhu YF, et al. The analysis on genetic evolution of Bian Chicken (*Gallus gallus*) population based on RAD-seq technology. Journal of Agricultural Biotechnology. 2019;27(12): 2198-2206. doi: 10.3969/j.issn.1674-7968.2019.12.011.

13. Sambrook J, Russell DW. Translated by Huang, PT 444 molecular cloning a laboratory manual (p. 445). Beijing, China: Science Press; 2002.

14. Kahnt B, Theodorou P, Soro A, Hollens-Kuhr H, Kuhlmann M, Pauw A, et al. Small and genetically highly structured populations in a long-legged bee, Rediviva longimanus, as inferred by pooled RAD-seq. BMC Evolutionary Biology. 2018;18(1): 1-12. doi: 10.1186/s12862-018-1313-z.

15. Baird NA, Etter PD, Atwood TS, Currey MC, Shiver AL, Lewis ZA, et al. Rapid SNP discovery and genetic mapping using sequenced RAD markers. PLoS ONE. 2008;3(10): e3376. doi: 10.1371/journal.pone.0003376.

16. Chen SF, Zhou YQ, Chen YR, Gu J. Fastp: An ultra-fast all-in-one FASTQ preprocessor. Bioinformatics. 2018;34(17): i884–i890. doi: 10.1093/bioinformatics/bty560.

17. Li H, Durbin R. Fast and accurate short read alignment with Burrows-Wheeler transform. Bioinformatics. 2009;25(14): 1754–1760. doi: 10.1093/bioinformatics/btp324.

18. Depristo MA, Banks E, Poplin R, Garimella KV, Maguire JR, Hartl C, et al. A framework for variation discovery and genotyping using next-generation DNA sequencing data. Nature Genetics. 2011;43(5): 491–498. doi: 10.1038/ng.806.

19. Pfeifer B, Wittelsbürger U, Ramos-Onsins SE, Lercher MJ. PopGenome: An efficient Swiss army knife for population genomic analyses in R. Molecular Biology and Evolution. 2014;31(7): 1929-1936. doi: 10.1093/molbev/msu136.

20. Su MK, Guo JF, Chen HB, Huang JC. F_{ST} statistical method for evaluating KIF1B gene rs17401966 polymorphism and heterogeneity source of hepatocellular carcinoma risk meta-analysis. International Journal of Laboratory Medicine. 2016;37(23): 3252-3254.

21. Gao Y, Yin SI, Wu L, Dai D, Wang H, Liu C, et al. Genetic diversity and structure of wild and cultivated Amorphophallus paeoniifolius populations in southwestern China as revealed by RAD- seq. Scientific Reports. 2017;7(1): 14183. doi: 10.1038/s41598-017-14738-6.

22. Li ZR. A systhetical application of principal component analysis and inheritance analysis in genetical space of wheat. Journal of Qinghai University. 1995;13(4): 38-42.

23. Yang J, Lee SH, Goddard ME, Visscher PM. GCTA: A tool for genome- wide complex trait analysis. The American Journal of Human Genetics. 2011;88(1): 76-82. doi: 10.1016/j.ajhg.2010.11.011.

24. Nei M, Li WH. Mathematical model for studying genetic variation in terms of restriction endonucleases. Proc Natl Acad Sci U S A. 1979;76(10): 5269-5273. doi: 10.1073/pnas.76.10.5269.

25. Hudson RR, Boos DD, Kaplan NL. A statistical test for detecting geographic subdivision. Molecular Biology and Evolution. 1992;9(1): 138-151. doi: 10.1093/oxfordjournals.molbev.a040703.

26. The R Core Team. R: A language and environment for statistical computing. 2.15.2 ed. 2012.

27. Ye J, Fang L, Zheng HK, Zhang Y, Chen J, Zhang ZJ, et al. WEGO: a web tool for plotting GO annotations. Nucleic Acids Res. 2006;34: 293-297. doi: 10.1093/nar/gkl031.

28. Mao X, Cai T, Olyarchuk JG, Wei L. Automated genome annotation and pathway identification using the KEGG Orthology (KO) as a controlled vocabulary. Bioinformatics. 2005;21: 3787–3793. doi: 10.1093/bioinformatics/bti430.

29. Wang FH, Bao HE, Odungrzil, Gao-Wa WY, Ertunmutu, Narembatu.

Analysis of biological characteristics of Alashan Bactrian camel based on whole genome resequencing. Journal of livestock ecology. 2019;40(8): 20-26.

30. Xiao YC, Jirimutu. Research progress of camel in drought resistance. Proceedings of the 5th China camel industry development conference. 2017; 223-227.

31. Subdengrizhil, Alima, Wuyunggeril, Lie Q, Zhang Y. Study on the living habits of Bactrian camel in Inner Mongolia. Journal of Heilongjiang Bayi Agricultural University. 2017;29(4): 49-53.

32. Anwar M, Zhang XL. The characteristics of ecological environment in Nanjiang region and its constraints on Urbanization. Journal of Southwest University (Natural Science Edition). 2011;33(4): 52-57.

33. Tian YZ, Nurbia U, Wang LJ, Wu WW, Xu XM, Zhang YH, et al. Genetic diversity analysis of Bactrian camel in six places of Xinjiang. Animal Husbandry & Veterinary Medicine. 2012;44(6): 38-43.

34. Wu XM, Yu F, Chen J, Su XZ, Li J. Construction of Dermal Papilla cDNA Library of Bovine Anagen Eyelash Follicle. Acta Agriculturae Boreali-occidentalis Sinica. 2007;5: 22-25.

35. Dai D, Li L, Huebner A, Zeng H, Guevara E, Claypool DJ, et al. Planar cell polarity effector gene Intu regulates cell fate-specific differentiation of keratinocytes through the primary cilia. Cell death and differentiation. 2013;20(1): 130-138. doi: 10.1038/cdd.2012.104.

36. Cao YW, Liu YX. Measures and breeding direction to promote the development of Sunite Bactrian camel breeding industry. Contemporary animal husbandry. 2014;24: 44-45.

37. Liang CL, Yang XX, Chi WZ, Liang DC, Wang XH. Study on the structure of Bactrian camel hair follicle group. Journal of Northwest Minorities University (Natural Science). 1994;1: 61-63.

38. Jamora C, Lee P, Kocieniewski P, Azhar M, Hosokawa R, Chai Y, et al. A signaling pathway involving TGF-beta2 and snail in hair follicle morphogenesis. PLoS biology. 2005;3(1): e11. doi: 10.1371/journal.pbio.0030011.

39. Wang JM, Cui PJ, Zhong YM, Li JW, Chu JM. Species richness pattern and its environmental interpretation in the plant region of Alashan Plateau. Journal of Beijing Forestry University. 2019;41(3): 14-23.

40. Wu S, Shi YJ, Mulligan P, Gay F, Landry J, Liu HF, et al. A YY1–INO80 complex regulates genomic stability through homologous recombination–based repair. Nature Structural & Molecular Biology. 2007;14(12): 1165-1172. doi: 10.1038/nsmb1332.

41. Azati, Hal A, Dillina. A survey of the variety resources of Mulei Bactrian camel in Xinjiang. China Animal Industry. 2012;20: 68-70. doi: 10.3969/j.issn.2095-2473.2012.20.027.

42. Koken MH, Reynolds P, Jaspers-Dekker I, Prakash L, Prakash S, Bootsma D, et al. Structural and functional conservation of two human

homologs of the yeast DNA repair gene RAD6. Proceedings of the National Academy of Sciences of the United States of America. 1991;88(20): 8865-8869. doi: 10.1073/pnas.88.20.8865.

Fig 1. Principal component analysis of seven domestic Bactrian camel populations in China.

Click here to access/download;Figure;Figure 1..tif

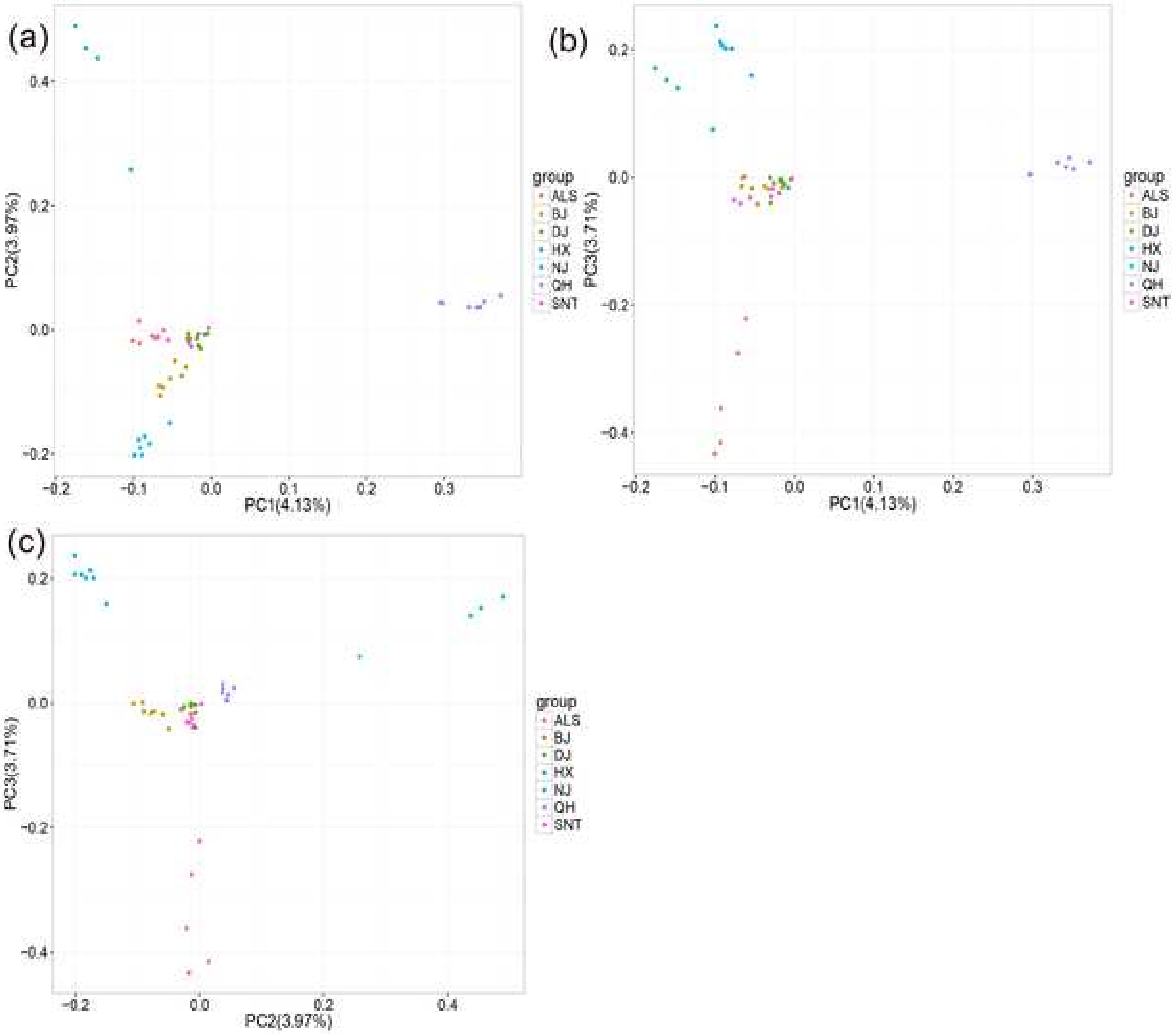

Fig 2. GO classification and KEGG enrichment of the selected genes of Nanjiang camels.

Click here to access/download;Figure;Figure 2..tif

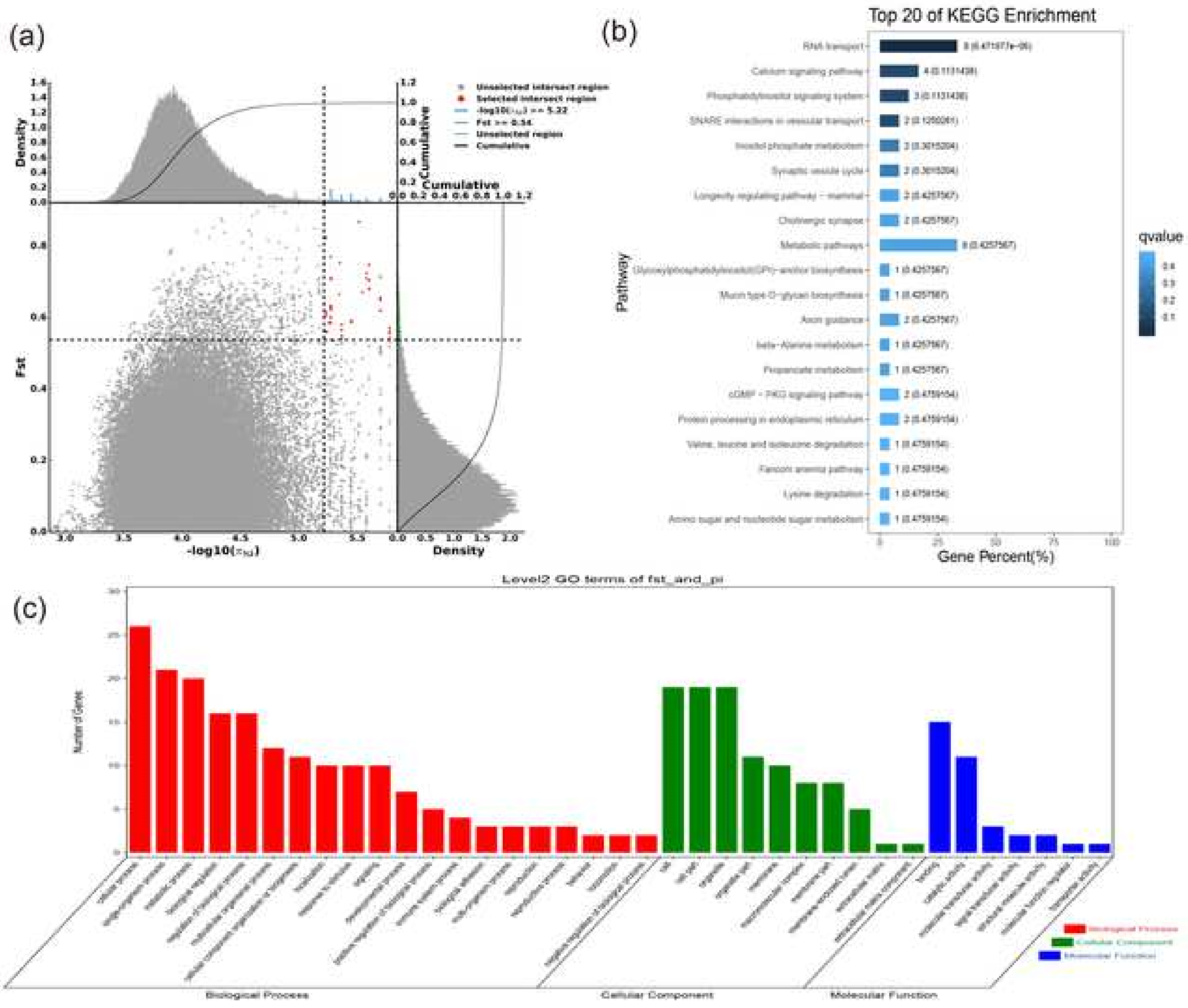

Fig 3. GO classification and KEGG enrichment of the selected genes of Sunite camels.

Click here to access/download;Figure;Figure 3..tif

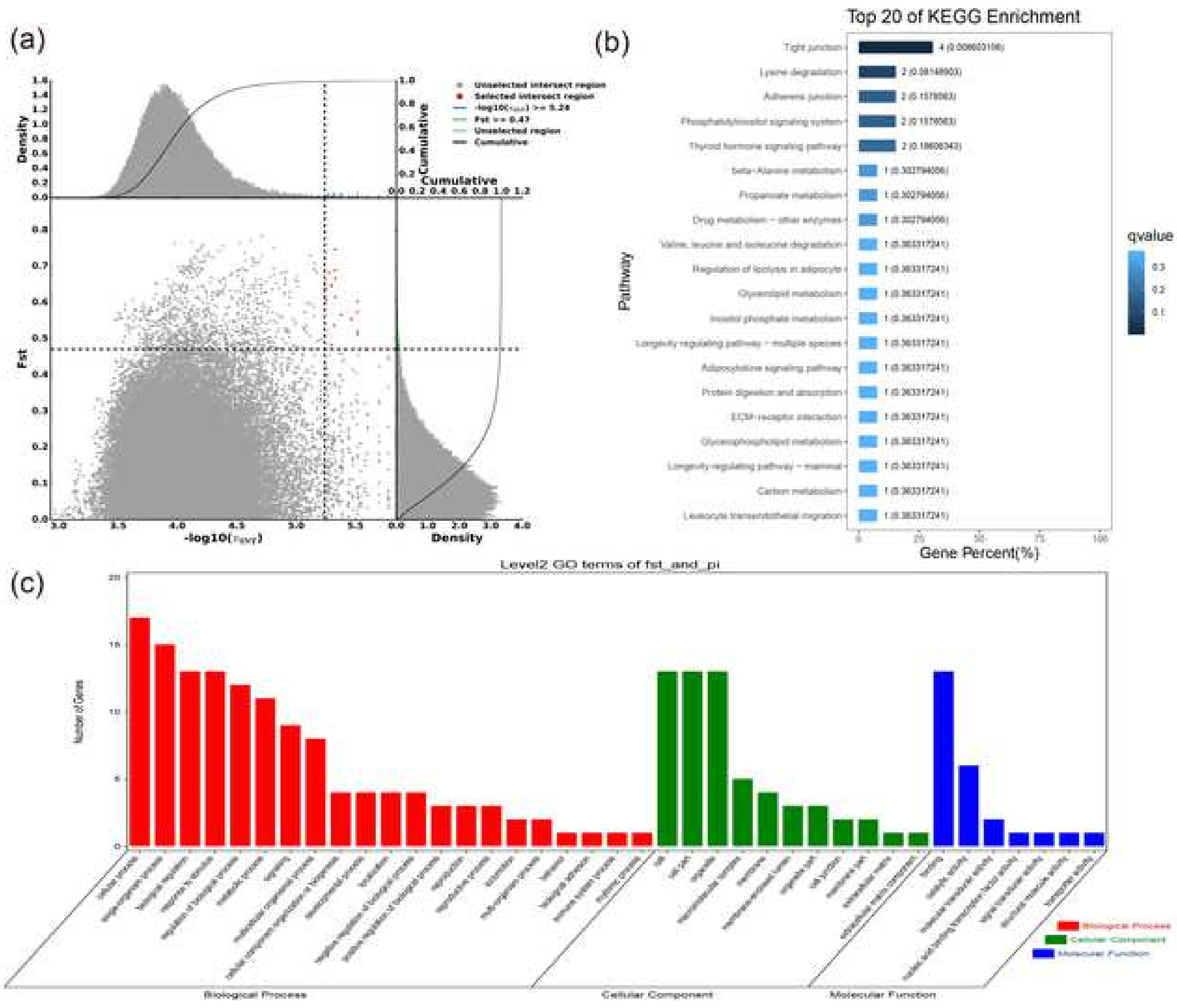

Fig 4. GO classification and KEGG enrichment of the selected genes of Alashan camels.

Click here to access/download;Figure;Figure 4..tif

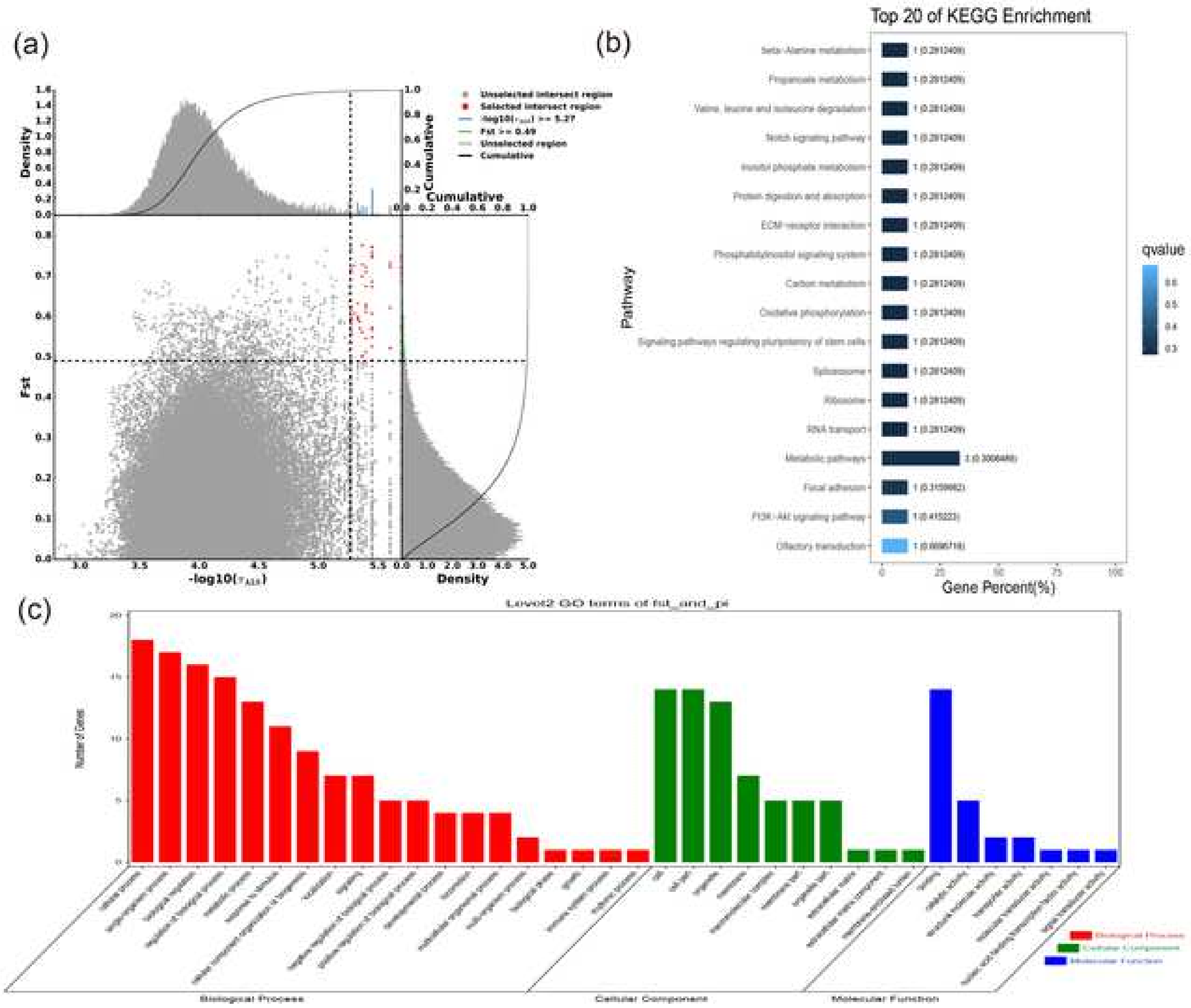

Fig 5. GO classification and KEGG enrichment of the selected genes of Dongjiang camels.

Click here to access/download;Figure;Figure 5..tif

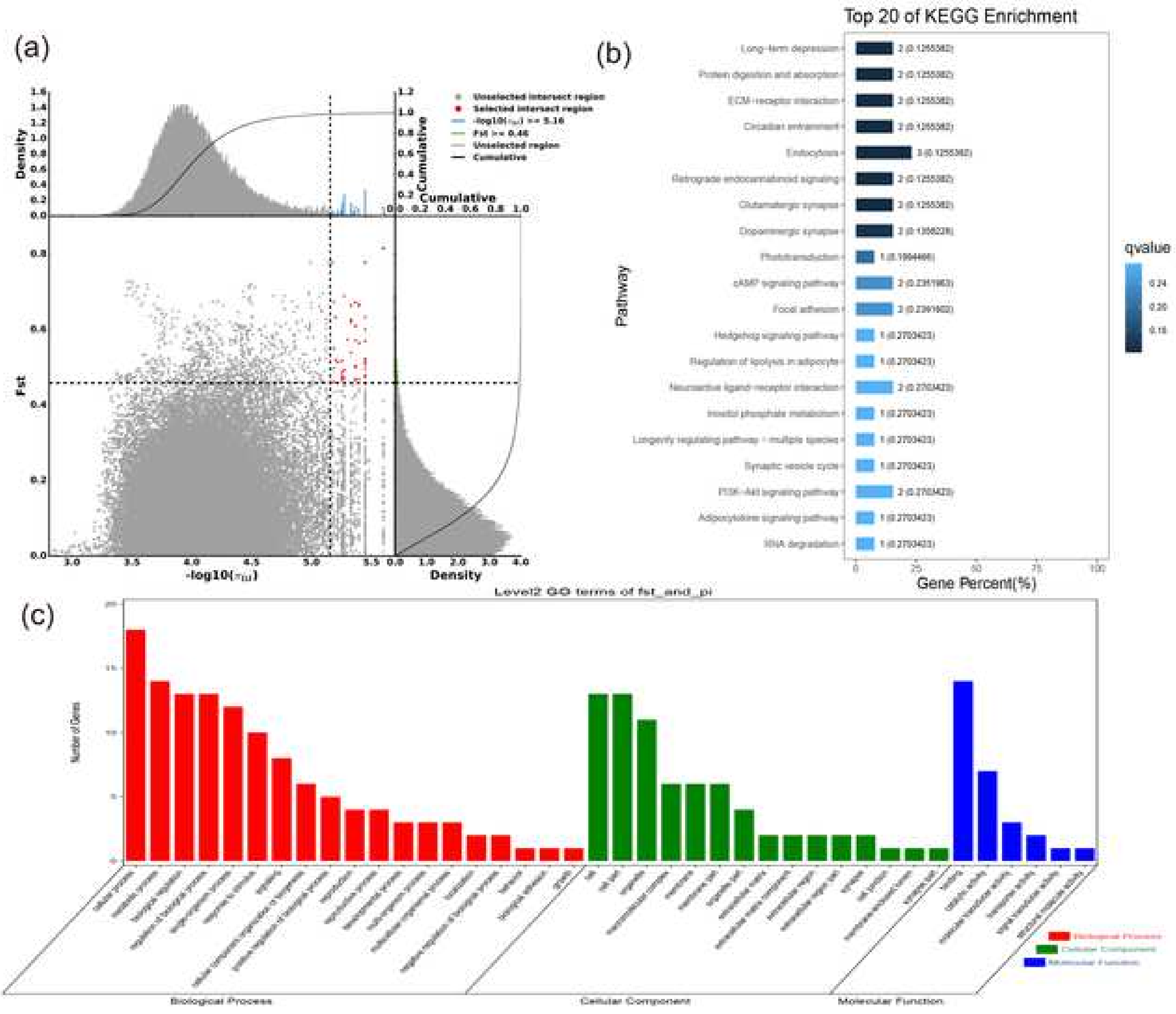

S1 Table. Sequencing results and quality filtering of reads.

Click here to access/download
Supporting Information
S1 Table..docx

S1 Fig. GO classification and KEGG enrichment of the selected genes of Beijiang camels.

Click here to access/download
Supporting Information
Figure S1..tif

S2 Fig. GO classification and KEGG enrichment of the selected genes of Qinghai camels.

Click here to access/download
Supporting Information
Figure S2..tif
