# Supplementary material for: Exploring the genomic resources of seven domestic Bactrian camel populations in China through restriction site-associated DNA sequencing
Source: PLoS One. 2021 Apr 29;16(4):e0250168. doi: 10.1371/journal.pone.0250168 (PMC8084232; doi:10.1371/journal.pone.0250168)
Supplement: Attachment — Submitted filename: Response to Reviewers.docx [file pone.0250168.s025.docx]

PONE-D-20-20388

Exploring the genomic resources of seven domestic Bactrian camel populations in China by restriction site-associated DNA sequencing

PLOS ONE

Dear Editor and Reviewers,

Thank you very much for the constructive comments made by the editor and reviewers to our manuscript “Exploring the genomic resources of seven domestic Bactrian camel populations in China by restriction site-associated DNA sequencing”. I have read all the comments carefully and answered each question one by one. Where appropriate we have also included the altered or additional text after this, shown in green font, as well as line numbers in the revised manuscript. We hope this is now suitable for publication in PLoS One. Once again, thank the editor and reviewers for their constructive comments. The main comments and our specific responses are detailed below.

Sincerely,

Chenmiao Liu

(On behalf of the co-authors)

**Journal Requirements:**

1. Please ensure that your manuscript meets PLOS ONE's style requirements, including those for file naming.

**Response:** Thank you very much for your review.

Has been revised.

I ensure that my manuscript meets PLOS ONE's style requirements, including those for file naming.

2. In your Methods, please describe the exact protocol used for blood sampling, including the volume of the samples and whether any analgesia was used.

**Response:** Thank you very much for your review.

Has been revised.

P5L94-97.

In total, 47 venous blood samples were collected (5 mL each). Miannaining was used during the sampling process, and each sample was derived from a different family; there was no kinship among individuals.

3. We suggest you thoroughly copyedit your manuscript for language usage, spelling, and grammar.

**Response:** Thank you very much for your review.

I have copyedited my manuscript thoroughly for language usage, spelling, and grammar.

**Reviewers' comments:**

1. Is the manuscript technically sound, and do the data support the conclusions?

**Response:** Thank you very much for your review.

Here, we used restriction site-associated DNA sequencing (RAD-seq) to detect single nucleotide polymorphism (SNP) markers in seven domestic Bactrian camel populations. The identified SNPs were further evaluated using fixation index (*F*_ST_) and principal component analysis (PCA). *F*_ST_ and PCA showed that Hexi camels were genetically distant from domestic Bactrian camels in other populations. Based on differences in geographical location, farming, and animal husbandry production areas/modes, with Hexi camels as the control population and Nanjiang, Sunite, Alashan, Dongjiang, Beijiang, and Qinghai camels as the selection populations for selection signature analysis. Gene Ontology (GO) classifications and Kyoto Encyclopedia of Genes and Genomes (KEGG) enrichment analyses were performed, and the functions of these genes were further studied by Genecards to identify the genes that may be related to the unique characteristics of the camel population, such as heat resistance and stress resistance.

2. Has the statistical analysis been performed appropriately and rigorously?

**Response:** Thank you very much for your review.

In this study, we explored genome-wide SNP markers of seven domestic Bactrian camel populations by RAD-seq. The identified SNPs were further evaluated using fixation index (*F*_ST_) and principal component analysis (PCA). Based on differences in geographical location, farming, and animal husbandry production areas/modes, with Hexi camels as the control population and Nanjiang, Sunite, Alashan, Dongjiang, Beijiang, and Qinghai camels as the selection populations for selection signature analysis. Gene Ontology (GO) classifications and Kyoto Encyclopedia of Genes and Genomes (KEGG) enrichment analyses were performed, and the functions of these genes were further studied by Genecards to identify the genes that may be related to the unique characteristics of the camel population, such as heat resistance and stress resistance.

3. Have the authors made all data underlying the findings in their manuscript fully available?

**Response:** Thank you very much for your review.

In total, 1,568,087 SNPs were genotyped. *F*_ST_ and PCA showed that Hexi camels were genetically distant from domestic Bactrian camels in other populations. Based on differences in geographical location, farming, and animal husbandry production areas/modes, with Hexi camels as the control population and Nanjiang, Sunite, Alashan, Dongjiang, Beijiang, and Qinghai camels as the selection populations for selection signature analysis, 54, 29, 42, 34, 32, and 21 selected genes were obtained, respectively. We also found that Nanjiang (*HSPA4L* and *INTU*), Sunite (*SNAI1*), Alashan (*INO80*), and Dongjiang camels (*UBE2A*) harbored genes related to the environment and characteristics.

4. Is the manuscript presented in an intelligible fashion and written in standard English?

**Response:** Thank you very much for your review.

Has been revised.

I have copyedited my manuscript thoroughly for language usage, spelling, and grammar.

**Review Comments to the Author:**

**Reviewer #1:**

1. In this manuscript, RAD-seq was used to detect SNP markers from seven distinct domestic Bactrian camel populations from China. SNPs were used for Fixation index (FST) and principal component (PCA) analysis, and involved genes were tested for possible selection, where their function was further studied. In my opinion, this manuscript is of importance to the scientific community and has great potential. Nevertheless, I have several suggestions and comments in which I believe it might improve the impact of it.
   **Response:** Thank you very much for your review. I have read all the suggestions and comments carefully and answered each question one by one.

**General comments and suggestions:**
1. In general, I feel that connection between sentences is missing throughout the whole text in order to create a better flow while reading. See, for example, l.50-55. I would suggest to review the whole manuscript and create better links between sentences as well as sentence construction. The discussion is also very descriptive and lacks connection between sentences (more comments about the discussion below).

**Response:** Thank you very much for your review.

Has been revised.

P3L46-53 and P14L288-P15L318.

These camels are also the source of various livestock products. For example, camel hair is a good textile material containing natural protein fiber [2]. Additionally, camel milk is rich in nutrition and has hypoglycemic and anticancer activities [3, 4]. Camel meat exhibits high moisture, high protein, low fat, and low cholesterol and has been shown to have medicinal and health-protective characteristics, suggesting applications in the treatment of some diseases [5-7].

Wensu County is close to the Taklimakan Desert. Nanjiang camels have been living in a natural environment with abundant light energy, dry climate with little rain, strong winds, and sand for a long time [32]. Thus, these camels show extremely strong heat resistance [33]. *HSPA4L* (encoding heat shock protein family A member 4 like) is a candidate gene related to heat resistance [29] and [cellular response to heat stress](http://pathcards.genecards.org/card/cellular_response_to_heat_stress). Thus, *HSPA4L* may be an interesting target for studying the heat resistance of Nanjiang camels. In addition, Nanjiang camels have 3–5-cm-long eyelashes; the length of the eyelashes is determined by the duration of its hair follicle growth period [34]. Inturned planar cell polarity protein (encoded by *INTU*) is involved in hair follicle morphogenesis [35]. Therefore, studies of *INTU* may provide insights into the distinct eyelash length of Nanjiang camels. Notably, Sunite camels have a thick and dense villus layer and many protective hairs, resulting in higher villus yields [36]. The key to increasing the villus yield is to ensure the full development of hair follicles [37]. *SNAI1* (encoding Snail family transcriptional repressor 1) is involved in hair follicle morphogenesis [38], suggesting that *SNAI1* may be an appropriate target for studying the villus yields of Sunite camels. The central production area of Alashan camels is located in the Alashan Plateau in the west of Inner Mongolia. The Alashan Plateau has strong ultraviolet rays, and Alashan camels exhibit anti-ultraviolet physiological functions, developed through a long-term evolution process [39]. *INO80* (encoding INO80 complex ATPase subunit) is involved in cellular response to UV [40]. *INO80* is therefore an interesting target for future experimental research. Mulei County is located in a temperate desert with strong ultraviolet rays and harsh natural ecological conditions. Dongjiang camels have also evolved physiological functions that can protect against ultraviolet radiation [41]. Because *UBE2A* (encoding ubiquitin-conjugating enzyme E2A) is involved in cellular response to UV [42], this gene may have applications in future studies of anti-ultraviolet radiation functions in these camel populations.

2. Some abbreviations for text comprehension have no explanation assuming all readers know what is relates to, for example l.59: MSY. I would suggest to review all abbreviations.

**Response:** Thank you very much for your review. I have explained and reviewed all abbreviations.

P3L62-65.

Felkel et al. [8] generated a 3.8-Mbp assembly of the male-specific part of the Y chromosome (MSY) of Bactrian camels based on short-read next-generation sequencing data.

3. The authors wrote several times in the different manuscript sections that FST and PCA were used for testing “phylogenetic” distances from domestic Bactrian camels in different populations. But the authors did not do any phylogenetic analysis, they have just tested for population differentiation (Fst) and individual-to-individual (PCA) genetic distances. I suggest the authors change it to “genetically” instead.

**Response:** Thank you very much for your review. I had changed “phylogenetic” to “genetically” instead.

4. At the beginning the taxonomic description of the domestic Bactrian camel is missing. Only in the line 59 it is stated the scientific name, but with no connection to the common name.

**Response:** Thank you very much for your review.

Has been revised.

P3L62-65.

Felkel et al. [8] generated a 3.8-Mbp assembly of the male-specific part of the Y chromosome (MSY) of Bactrian camels based on short-read next-generation sequencing data.

5. I don’t understand why the authors justify the use of Hexi camel population as “control population” for selection tests just because “these camels are genetically more distant to the other populations”, to then infer environmental pressures or production traits. I am not saying it is a bad choice, I am just commenting and calling attention to the fact that there is context information missing to understand the thought behind it. Why did the authors choose these populations specifically? What do they have in common and what do they have that differentiates them? Do all populations except for Hexi live under the same environmental conditions? Are the animals under different selective pressures on production traits on the control population compared to the others? It is always very interesting to assess genes under selection, but it is also very important to contextualize the results with the real situation, in this case, contextualize the results with the different camel populations the authors are using. I comment more on this in the “introduction” and “discussion” detailed suggestions where I suggest less descriptive text on the discussion and more information on these populations (maybe this information should start in the introduction section) to better understand the whole manuscript message.

**Response:** Thank you very much for your review.

Has been revised.

P1L20-P2L25 and P3L54-62.

Based on differences in geographical location, farming, and animal husbandry production areas/modes, with Hexi camels as the control population and Nanjiang, Sunite, Alashan, Dongjiang, Beijiang, and Qinghai camels as the selection populations for selection signature analysis, 54, 29, 42, 34, 32, and 21 selected genes were obtained, respectively.

Seven domestic Bactrian camel populations are located in different production areas. The production area of Hexi camels is dominated by the farming culture of the Han nationality, whereas the production area of other domestic Bactrian camels is dominated by minority nomadic cultures. In addition, Hexi camels are located in the northern foot of the Qilian Mountains, close to the Badain Jaran Desert, and are separated from other domestic Bactrian camel populations based on geographical location. Therefore, Hexi camels can be used as a control group for exploration of the genomic resources of this species.

**Detailed comments and suggestions:**

**Abstract.**

6. L15-17: “In this study, restriction site associated DNA sequencing (RAD-seq) was used to detect SNP markers of the seven domestic Bactrian camel populations.” There are not only seven Bactrian populations in China, so I would suggest to delete “the”. Also, it is “Restriction site-associated DNA sequencing” (hyphen missing).

**Response:** Thank you very much for your review.

Has been revised.

P1L13-16.

Here, we used restriction site-associated DNA sequencing (RAD-seq) to detect single nucleotide polymorphism (SNP) markers in seven domestic Bactrian camel populations.

7. L17-20: “The filtered SNPs were used for Fixation index (FST) analysis and principal component analysis (PCA) to find out the population which is phylogenetically distant from domestic Bactrian camels in other populations as the control population.” I suggest to rephrase this sentence.

**Response:** Thank you very much for your review.

Has been revised.

P1L16-20.

The identified SNPs were further evaluated using fixation index (*F*_ST_) and principal component analysis (PCA). *F*_ST_ and PCA showed that Hexi camels were genetically distant from domestic Bactrian camels in other populations.

**Introduction.**
8. L55-57: “In addition, the domestic Bactrian camel lives in hot and arid desert environment, which has potential ecological adaptation value.” Domestic Bactrian camels do not live exclusively in hot and arid, but also in cold and high altitudes. I would suggest to rephrase this sentence or to justify where specifically they live in these conditions – again, it would help if there would be already here more information on the different populations that were chosen in this study.

**Response:** Thank you very much for your review.

Has been revised.

P3L54-58.

Seven domestic Bactrian camel populations are located in different production areas. The production area of Hexi camels is dominated by the farming culture of the Han nationality, whereas the production area of other domestic Bactrian camels is dominated by minority nomadic cultures.

9. L. 58: delete “(2019)”.

**Response:** Thank you very much for your review.

Has been deleted.

10. L. 59: “Camelus bactrianus” should be in italic.

**Response:** Thank you very much for your review.

Has been revised.

P3L62-65.

Felkel et al. [8] generated a 3.8-Mbp assembly of the male-specific part of the Y chromosome (MSY) of Bactrian camels based on short-read next-generation sequencing data.

11. L58-66: The authors start by telling about this other study (in the 3rd person), and then on the l.63 they state “in the previous study, we used…”. I would suggest to be coherent and either write it in the 1st or 3rd person.

**Response:** Thank you very much for your review.

Has been revised.

P4L68-72.

Additionally, in a previous study, Liu et al. [9] used SNPs to study the genetic diversity and genetic structure of seven domestic Bactrian camel populations; in this study, the phylogenetic relationships of these populations were clarified, laying a foundation for protecting their biodiversity.

12. L70-71: “RAD-seq analysis is becoming more and more perfect” I suggest to rephrase “more and more perfect”.

**Response:** Thank you very much for your review.

Has been revised.

P4L75-77.

With the establishment of high-throughput sequencing technology and bioinformatics technology, RAD-seq analysis has become more refined and has been applied to many organisms [10].

13. L71-78: It is good that the authors state examples of studies that used the same technique in different species like eggplant or chicken, but I would suggest to state examples closer to camels or even within camelid species, that have used the same (RADseq) technique.

**Response:** Thank you very much for your review.

Has been revised.

P4L78-82.

For example, Li et al. [11] used RAD-seq for genome SNP typing of 618 sows to achieve accurate prediction of genome breeding value and evaluated the accuracy of breeding value prediction. Moreover, Wang et al. [12] successfully employed RAD-seq to explore genome-wide SNPs among six breeds of Sichuan cattle.

14. L82-87: Please add citations or links to “GO”, “KEGG” and “Genecards”.

**Response:** Thank you very much for your review.

Has been revised.

P4L83-L87.

In this study, we explored genome-wide SNP markers of seven domestic Bactrian camel populations by RAD-seq, using Hexi camels as the control group for selection signature analysis. We aimed to identify genes that may be related to the unique characteristics of these camels, such as heat resistance and stress resistance.

15. Missing a more complete sentence in the end of the introduction with the aim and importance of this study as right now these are not very clearly stated.

**Response:** Thank you very much for your review.

Has been revised.

P4L87-P5L90.

Our results established interesting targets for subsequent genomics research of the domestic Bactrian camel and may facilitate genetic association analysis of economically important traits of this species.

**Materials and Methods.**
16. L. 123-128: substitute “readings” to “reads”.

**Response:** Thank you very much for your review.

Has been revised.

P6L126-P7L131.

Raw reads were processed to obtain high-quality clean reads using three strict filtering standards, as follows: (1) removing reads with greater than or equal to 10% unidentified nucleotides (N), (2) removing reads with greater than 50% of bases having phred quality scores of less than or equal to 20, and (3) removing reads aligned to the barcode adapter.

17. The authors did SNP filtering, but did the authors do sample filtering (e.g. filtering samples out with missing call of a specific rate)?

**Response:** Thank you very much for your review.

We didn't find abnormal samples in the process of analysis, and the variation information of each sample was generally consistent, so we didn't filter the samples.

| sample | snp | indel |
| --- | --- | --- |
| ALS-1 | 400909 | 59293 |
| ALS-2 | 431342 | 65091 |
| ALS-3 | 416516 | 62291 |
| ALS-4 | 395045 | 59031 |
| ALS-5 | 378303 | 56586 |
| ALS-6 | 376948 | 55913 |
| ALS-7 | 238629 | 33650 |
| BJ-1 | 351434 | 52414 |
| BJ-2 | 347812 | 51443 |
| BJ-3 | 333301 | 48864 |
| BJ-4 | 348118 | 51857 |
| BJ-5 | 302660 | 44435 |
| BJ-6 | 291205 | 42055 |
| BJ-7 | 212065 | 30470 |
| DJ-1 | 293884 | 43115 |
| DJ-2 | 412285 | 61374 |
| DJ-3 | 274259 | 39590 |
| DJ-4 | 384356 | 56215 |
| DJ-5 | 510888 | 76466 |
| DJ-6 | 423925 | 62800 |
| DJ-7 | 412384 | 61186 |
| HX-1 | 450250 | 68562 |
| HX-2 | 434519 | 65152 |
| HX-3 | 405875 | 60727 |
| HX-4 | 433282 | 64685 |
| HX-5 | 358705 | 52764 |
| NJ-1 | 298725 | 42553 |
| NJ-2 | 339008 | 49262 |
| NJ-3 | 262918 | 37763 |
| NJ-4 | 346445 | 50596 |
| NJ-5 | 395494 | 58363 |
| NJ-6 | 447244 | 67461 |
| NJ-7 | 283301 | 40999 |
| QH-1 | 454624 | 68257 |
| QH-2 | 410231 | 60948 |
| QH-3 | 466470 | 70295 |
| QH-4 | 387525 | 57182 |
| QH-5 | 403445 | 58735 |
| QH-6 | 379883 | 56187 |
| QH-7 | 403622 | 59955 |
| SNT-1 | 430952 | 64525 |
| SNT-2 | 353668 | 52768 |
| SNT-3 | 342415 | 50452 |
| SNT-4 | 331900 | 47946 |
| SNT-5 | 437473 | 65791 |
| SNT-6 | 390642 | 58472 |
| SNT-7 | 312674 | 45327 |

18. L. 154: are the “internal PERL scripts” available to the scientific community, to promote transparency?

**Response:** Thank you very much for your review.

The “internal PERL scripts” is available in Supporting information.

S1 File. The in-house PERL scripts. (DOCX)

19. I think it would be interesting to see ADMIXTURE analysis in order to infer population structure and for calculating ancestry estimates.

**Response:** Thank you very much for your review.

ADMIXTURE analysis in order to infer population structure and for calculating ancestry estimates is available in the literature.

Liu CM, Chen HL, Ren ZJ, Zhang CD, Yang XJ. Population genetic analysis of the domestic Bactrian camel in China by RAD-seq. Ecology and evolution. 2019;34(4): 11232-11242. [doi: 10.1002/ece3.5624](https://doi.org/10.1002/ece3.5624).

**Results.**

20. L. 179: I suggest to stated with how many SNP the analysis were performed.

**Response:** Thank you very much for your review.

Has been revised.

P9L184-186.

In total, 1,568,087 SNPs were identified in this study. After removing sites with a missing rate of 50% or more, the remaining 865,774 loci were used for *F*_ST_ and PCA.

21. L. 186: The respective initials of the populations to the populations in the table description are not stated.

**Response:** Thank you very much for your review.

Has been revised.

P10L194-196.

BJ, Beijiang camels; DJ, Dongjiang camels; HX, Hexi camels; NJ, Nanjiang camels; QH, Qinghai camels; SNT, Sunite camels; ALS, Alashan camels.

22. L. 191: please make reference to the figure number.

**Response:** Thank you very much for your review.

Has been revised.

P10L203-206.

Based on the last two PCs (PC2 = 3.97%, PC3 = 3.71%), the Hexi, Nanjiang, and Alashan camels clustered together separately and were genetically distant from the domestic Bactrian camels in other populations (Fig 2).

**Discussion.**

23. L. 264: I would suggest to change “In the study” to “In this study”.

**Response:** Thank you very much for your review.

Has been revised.

P13L268-269.

In this study, 1,568,087 SNP markers from seven domestic Bactrian camel populations were detected by RAD-seq.

24. L. 266: Fst in italic.

**Response:** Thank you very much for your review.

Has been revised.

P13L269-271.

The filtered SNPs were used for *F*_ST_ and PCA to determine the population that was genetically distant from the domestic Bactrian camel in other populations.

25. I really feel it is missing connection between the results and the camel populations history. I think the manuscript would improve greatly if the authors would change from a descriptive discussion to a more “story telling” format around the subject. This means that maybe there should be a bit more contextualization of these populations first (starting already in the introduction), and then relate to their findings, instead of just being very descriptive in major results. This would be very helpful to better understand their findings. This also connects to one of my comments on creating better flow between sentences.

**Response:** Thank you very much for your review.

Has been revised.

P3L54-62 and P14L288-P15L318.

Seven domestic Bactrian camel populations are located in different production areas. The production area of Hexi camels is dominated by the farming culture of the Han nationality, whereas the production area of other domestic Bactrian camels is dominated by minority nomadic cultures. In addition, Hexi camels are located in the northern foot of the Qilian Mountains, close to the Badain Jaran Desert, and are separated from other domestic Bactrian camel populations based on geographical location. Therefore, Hexi camels can be used as a control group for exploration of the genomic resources of this species.

Wensu County is close to the Taklimakan Desert. Nanjiang camels have been living in a natural environment with abundant light energy, dry climate with little rain, strong winds, and sand for a long time [32]. Thus, these camels show extremely strong heat resistance [33]. *HSPA4L* (encoding heat shock protein family A member 4 like) is a candidate gene related to heat resistance [29] and [cellular response to heat stress](http://pathcards.genecards.org/card/cellular_response_to_heat_stress). Thus, *HSPA4L* may be an interesting target for studying the heat resistance of Nanjiang camels. In addition, Nanjiang camels have 3–5-cm-long eyelashes; the length of the eyelashes is determined by the duration of its hair follicle growth period [34]. Inturned planar cell polarity protein (encoded by *INTU*) is involved in hair follicle morphogenesis [35]. Therefore, studies of *INTU* may provide insights into the distinct eyelash length of Nanjiang camels. Notably, Sunite camels have a thick and dense villus layer and many protective hairs, resulting in higher villus yields [36]. The key to increasing the villus yield is to ensure the full development of hair follicles [37]. *SNAI1* (encoding Snail family transcriptional repressor 1) is involved in hair follicle morphogenesis [38], suggesting that *SNAI1* may be an appropriate target for studying the villus yields of Sunite camels. The central production area of Alashan camels is located in the Alashan Plateau in the west of Inner Mongolia. The Alashan Plateau has strong ultraviolet rays, and Alashan camels exhibit anti-ultraviolet physiological functions, developed through a long-term evolution process [39]. *INO80* (encoding INO80 complex ATPase subunit) is involved in cellular response to UV [40]. *INO80* is therefore an interesting target for future experimental research. Mulei County is located in a temperate desert with strong ultraviolet rays and harsh natural ecological conditions. Dongjiang camels have also evolved physiological functions that can protect against ultraviolet radiation [41]. Because *UBE2A* (encoding ubiquitin-conjugating enzyme E2A) is involved in cellular response to UV [42], this gene may have applications in future studies of anti-ultraviolet radiation functions in these camel populations.

26. L.284-285: Please elaborate on how this finding fills in the knowledge gap.

**Response:** Thank you very much for your review.

Has been revised.

P14L284-287.

Overall, our findings established interesting targets for genetic association analysis of important traits of the domestic Bactrian camel in China and provided a basis for genome studies in this species [29–31].

**Reviewer #2:**

Manuscript PONE-D-20-20388 seemed to be designed, based on the same RAD-seq data reported before by the same group of authors (Liu CM, Chen HL, Ren ZJ, Zhang CD, Yang XJ. Population genetic analysis of the domestic Bactrian camel in China by RAD-seq. Ecology and evolution. 2019;34(4): 11232-11242. doi: 10.1002/ece3.5624.), to examine the population differentiation and then selection signals of the seven Chinese Bactrian camels. After reading their early article published in 2019, it is hard to see the value of publishing this manuscript with limited new result.
**Response:** Thank you very much for your review.

This study and early article published in 2019 reflected different analysis content and purpose. The early article studied the genetic diversity and genetic structure of the domestic Bactrian camel, and conducted population genetic analysis of the species. In this study, based on differences in geographical location, farming, and animal husbandry production areas/modes, with Hexi camels as the control population and Nanjiang, Sunite, Alashan, Dongjiang, Beijiang, and Qinghai camels as the selection populations for selection signature analysis. We found that Nanjiang (*HSPA4L* and *INTU*), Sunite (*SNAI1*), Alashan (*INO80*), and Dongjiang camels (*UBE2A*) harbored genes related to the environment and characteristics. Our results established interesting targets for subsequent genomics research of the domestic Bactrian camel and may facilitate genetic association analysis of economically important traits of this species.

1. The manuscript is written in very poor English.

**Response:** Thank you very much for your review.

Has been revised.

I have copyedited my manuscript thoroughly for language usage, spelling, and grammar.
2. The data and major analyses including FST and principle component analyses were already included in the article published by the same authors.

**Response:** Thank you very much for your review.

In the early article, we calculated the average *F*_ST_ of each population. In this study, we presented the *F*_ST_ between the two populations.

In this study, we identified the population which was far away from other domestic Bactrian camels in genetically based on different PCs (PC1=4.13%, PC2=3.97%, PC3=3.71%).
3. The selection signal analyses based on the π value and FST values from the comparison of the Hexi camel population with the other six camel populations make almost no sense. Considering the very low number of samples and also genetic admixture of the Hexi camels with Dongjiang, Qinghai or Sunite camels as shown in the article of 2019 and also the genetic differentiation of the Qinghai camels at PC1 in this manuscript, it is not justified to treat Hexi camels as a control population. Therefore, the annotations of the what so called functional genes are not reliable at all.

**Response:** Thank you very much for your review.

Seven domestic Bactrian camel populations are located in different production areas. The production area of Hexi camels is dominated by the farming culture of the Han nationality, whereas the production area of other domestic Bactrian camels is dominated by minority nomadic cultures. In addition, Hexi camels are located in the northern foot of the Qilian Mountains, close to the Badain Jaran Desert, and are separated from other domestic Bactrian camel populations based on geographical location. Therefore, Hexi camels can be used as a control group for exploration of the genomic resources of this species.
