# Supplementary material for: Exploring the genomic resources of seven domestic Bactrian camel populations in China through restriction site-associated DNA sequencing
Source: PLoS One. 2021 Apr 29;16(4):e0250168. doi: 10.1371/journal.pone.0250168 (PMC8084232; doi:10.1371/journal.pone.0250168)
Supplement: Attachment — Submitted filename: Response to Reviewers.docx [file pone.0250168.s026.docx]

PONE-D-20-20388R1
Exploring the genomic resources of seven domestic Bactrian camel populations in China by restriction site-associated DNA sequencing
PLOS ONE

Dear Editor and Reviewers,

Thank you very much for the constructive comments made by the editor and reviewers to our manuscript “Exploring the genomic resources of seven domestic Bactrian camel populations in China by restriction site-associated DNA sequencing”. I have read all the comments carefully and answered each question one by one. Where appropriate we have also included the altered or additional text after this, shown in green font, as well as line numbers in the revised manuscript. We hope this is now suitable for publication in PLoS One. Once again, thank the editor and reviewers for their constructive comments. The main comments and our specific responses are detailed below.

Sincerely,

Chenmiao Liu

(On behalf of the co-authors)

**Comments to the Author**

1. If the authors have adequately addressed your comments raised in a previous round of review and you feel that this manuscript is now acceptable for publication, you may indicate that here to bypass the “Comments to the Author” section, enter your conflict of interest statement in the “Confidential to Editor” section, and submit your "Accept" recommendation.

**Response:** Thank you very much for your review.

I have read all the comments carefully and answered each question one by one. I hope the manuscript is suitable for publication on *PLoS One*.

2. Is the manuscript technically sound, and do the data support the conclusions?
The manuscript must describe a technically sound piece of scientific research with data that supports the conclusions. Experiments must have been conducted rigorously, with appropriate controls, replication, and sample sizes. The conclusions must be drawn appropriately based on the data presented.

**Response:** Thank you very much for your review.

I'm sorry about that.

P1l20-P2l25 and P3l54-l62.

① Based on differences in farming and animal husbandry production areas/modes, the production area of Hexi camels is dominated by the farming culture of the Han nationality, whereas the production area of other domestic Bactrian camels is dominated by minority nomadic cultures. Hexi camels and other domestic Bactrian camels have different production areas and modes, resulting in the selection direction differences between Hexi camels and other domestic Bactrian camels.

② According to differences in geographical location, Hexi camels are located in the northern foot of the Qilian Mountains, close to the Badain Jaran Desert, and are separated from other domestic Bactrian camel populations based on geographical location.

③ In the original project design, we used the dromedary (Camelus dromedarius) as the outgroup. However, it is difficult to find dromedary in China at present. In the next step of research, we will actively seek cooperation with foreign countries to collect camel blood samples along the Silk Road to make up for the deficiencies of current research.

3. Has the statistical analysis been performed appropriately and rigorously?

**Response:** Thank you very much for your review.

4. Have the authors made all data underlying the findings in their manuscript fully available?

**Response:** Thank you very much for your review.

5. Is the manuscript presented in an intelligible fashion and written in standard English?

**Response:** Thank you very much for your review.

We have used Editage professional scientific editing service to make the changes. I hope the manuscript meets the requirements of *PLoS One*.

**Review Comments to the Author**

Reviewer #1: In general, the authors have tried to address my suggestions, but not all of them. I can see an improvement on sentence connection, but I am not sure if this was enough to improve the manuscript in general. I feel the authors could have taken more time to analyse and answer to each of my suggestions, and either apply them or justify their choices. Also, I still cannot understand - and it was also mentioned by me on the first review - why the authors have chosen the Hexi population as a control population. Unfortunately, in a meaningful biological way, the justifications are not enough for a proper scientific understanding, and this part being their main finding (selection), it has to be improved.

**Response:** Thank you very much for your review.

**(1)** We have used Editage professional scientific editing service to make the changes. I hope the manuscript meets the requirements of *PLoS One*.

**(2)** I'm sorry about that.

P1l20-P2l25 and P3l54-l62.

① Based on differences in farming and animal husbandry production areas/modes, the production area of Hexi camels is dominated by the farming culture of the Han nationality, whereas the production area of other domestic Bactrian camels is dominated by minority nomadic cultures. Hexi camels and other domestic Bactrian camels have different production areas and modes, resulting in the selection direction differences between Hexi camels and other domestic Bactrian camels.

② According to differences in geographical location, Hexi camels are located in the northern foot of the Qilian Mountains, close to the Badain Jaran Desert, and are separated from other domestic Bactrian camel populations based on geographical location.

③ In the original project design, we used the dromedary (Camelus dromedarius) as the outgroup. However, it is difficult to find dromedary in China at present. In the next step of research, we will actively seek cooperation with foreign countries to collect camel blood samples along the Silk Road to make up for the deficiencies of current research.
