# Supplementary material for: Exploring the genomic resources of seven domestic Bactrian camel populations in China through restriction site-associated DNA sequencing
Source: PLoS One. 2021 Apr 29;16(4):e0250168. doi: 10.1371/journal.pone.0250168 (PMC8084232; doi:10.1371/journal.pone.0250168)
Supplement: Attachment — Submitted filename: Response to Reviewers.docx [file pone.0250168.s027.docx]

PONE-D-20-20388R4
Exploring the genomic resources of seven domestic Bactrian camel populations in China through restriction site-associated DNA sequencing
PLOS ONE

Dear Editor and Reviewer,

Thank you very much for the constructive comments made by the editor and reviewer to our manuscript “Exploring the genomic resources of seven domestic Bactrian camel populations in China through restriction site-associated DNA sequencing”. I have read all the comments carefully and answered each question one by one. Where appropriate we have also included the altered or additional text after this, shown in red font, as well as line numbers in the revised manuscript. We hope this is now suitable for publication in *PLoS One*. Once again, thank the editor and reviewer for their constructive comments. The main comments and our specific responses are detailed below.

Sincerely,

Chenmiao Liu

(On behalf of the co-authors)

**Comments to the Author**
1. If the authors have adequately addressed your comments raised in a previous round of review and you feel that this manuscript is now acceptable for publication, you may indicate that here to bypass the “Comments to the Author” section, enter your conflict of interest statement in the “Confidential to Editor” section, and submit your "Accept" recommendation.

Reviewer #4: (No Response)

**Response:** Thank you very much for your review.

I have read all the comments carefully and answered each question one by one. I hope the manuscript is suitable for publication on *PLoS One*.

2. Is the manuscript technically sound, and do the data support the conclusions?

The manuscript must describe a technically sound piece of scientific research with data that supports the conclusions. Experiments must have been conducted rigorously, with appropriate controls, replication, and sample sizes. The conclusions must be drawn appropriately based on the data presented.

Reviewer #4: No

**Response:** Thank you very much for your review.

Has been revised.

P1l14-18 and P3l49-52.

The pool of all remaining others were selected as the reference population, and the Nanjiang, Sunite, Alashan, Dongjiang, Beijiang, Qinghai, and Hexi camels were the target populations for selection signature analysis. We obtained 603, 494, 622, 624, 444, 588, and 762 selected genes, respectively, from members of the seven target populations.

Nanjiang camels have stronger heat resistance than the pool of all remaining others, Alashan camels form stronger anti-ultraviolet physiological functions than the pool of all remaining others. Thus, the pool of all remaining others can be used as a reference group for exploration of the genomic resources of their species.

3. Has the statistical analysis been performed appropriately and rigorously?

Reviewer #4: No

**Response:** Thank you very much for your review.

P1l14-18, P3l49-52 and P8l154-157.

The pool of all remaining others were selected as the reference population, and the Nanjiang, Sunite, Alashan, Dongjiang, Beijiang, Qinghai, and Hexi camels were the target populations for selection signature analysis. We obtained 603, 494, 622, 624, 444, 588, and 762 selected genes, respectively, from members of the seven target populations.

Nanjiang camels have stronger heat resistance than the pool of all remaining others, Alashan camels form stronger anti-ultraviolet physiological functions than the pool of all remaining others. Thus, the pool of all remaining others can be used as a reference group for exploration of the genomic resources of their species.

Using the pool of all remaining others as the reference population and Nanjiang, Sunite, Alashan, Dongjiang, Beijiang, Qinghai, and Hexi camels as the target populations, 603, 494, 622, 624, 444, 588, and 762 selected genes, respectively, were obtained.

4. Have the authors made all data underlying the findings in their manuscript fully available?
Reviewer #4: Yes

**Response:** Thank you very much for your review.

5. Is the manuscript presented in an intelligible fashion and written in standard English?
Reviewer #4: Yes

**Response:** Thank you very much for your review.

**Reviewer #4: Major**
1) I agree with the Reviewer#3 that choosing Hexi camels as the reference for selection analysis is not appropriate. Even if Hexi camels are geographically, genetically and phenotypically different from other camels, there is no evidence that the other populations are derived from Hexi camels. I think a better strategy is to compare each population with the pool of all remaining others.

**Response:** Thank you very much for your review.

Has been revised. We chose a better strategy is to compare each population with the pool of all remaining others.

P1l14-18 and P3l49-52.

The pool of all remaining others were selected as the reference population, and the Nanjiang, Sunite, Alashan, Dongjiang, Beijiang, Qinghai, and Hexi camels were the target populations for selection signature analysis. We obtained 603, 494, 622, 624, 444, 588, and 762 selected genes, respectively, from members of the seven target populations.

Nanjiang camels have stronger heat resistance than the pool of all remaining others, Alashan camels form stronger anti-ultraviolet physiological functions than the pool of all remaining others. Thus, the pool of all remaining others can be used as a reference group for exploration of the genomic resources of their species.
2) In regard to the SNP quality, I think an independent dataset is needed to justify the authors’ filtering strategy. In fact, there are a lot of WGS data for domesticated Bactrian camels (e.g. doi: 10.1038/s42003-019-0734-6). It is surprising that the authors did not mention any recent research progress of the camel genome.

**Response:** Thank you very much for your review.

Has been revised.

(1) SNPs were re-filtered in accordance with the requirements of the last reviewer. In addition, we also refered to the related literature published on *PLoS One*.

Basak M, Uzun B, Yol E. Genetic diversity and population structure of the Mediterranean sesame core collection with use of genome-wide SNPs developed by double digest RAD-Seq. PLoS One. 2019;14(10): e0223757. doi: 10.1371/journal.pone.0223757.

Ren AY, Du K, Jia XB, Yang R, Wang J, Chen SY, et al. Genetic diversity and population structure of four Chinese rabbit breeds. PLoS One. 2019;14(9): e0222503. doi: 10.1371/journal.pone.0222503.

(2) At present, some studies have been conducted on domestic Bactrian camels. Ming et al. [8] sequenced the whole genome of 128 camels across Asia and revealed origin and migration of domestic Bactrian camels.

3) P3:51-54 The authors listed many phenotype differences among the populations such as heat resistance, villus yields, but there is no data or reference support the claims.

**Response:** Thank you very much for your review.

Has been revised.

Nanjiang camels have stronger heat resistance than the pool of all remaining others, Alashan camels form stronger anti-ultraviolet physiological functions than the pool of all remaining others[8, 9].

**8.** Tian YZ, Nurbia U, Wang LJ, Wu WW, Xu XM, Zhang YH, et al. Genetic diversity analysis of Bactrian camel in six places of Xinjiang. Animal Husbandry & Veterinary Medicine. 2012;44(6): 38-43.

**9.** Wang JM, Cui PJ, Zhong YM, Li JW, Chu JM. Species richness pattern and its environmental interpretation in the plant region of Alashan Plateau. Journal of Beijing Forestry University. 2019;41(3): 14-23.

**Minor**
Text in figures is not friendly to read. For example, what does “-log10({\pi_{N}})” mean?

**Response:** Thank you very much for your review.

Text in figures has been revised.
